# Health Technologies and Infrastructures for Supporting Home-Based Pediatric Palliative Care: Scoping Review

**DOI:** 10.2196/70687

**Published:** 2025-12-08

**Authors:** Simen Alexander Steindal, Anette Winger, Kirsti Riiser, Erik Bjørnerud, Weiqin Chen, Heidi Holmen

**Affiliations:** 1 VID Specialized University Oslo Norway; 2 Lovisenberg Diaconal University College Oslo Norway; 3 Department of Nursing and Health Promotion OsloMet—Oslo Metropolitan University Oslo Norway; 4 Department of Rehabilitation Science and Health Technology OsloMet—Oslo Metropolitan University Oslo Norway; 5 Faculty of Teacher Education and Languages Ostfold University College Halden Norway; 6 Department of Computer Science OsloMet—Oslo Metropolitan University Oslo Norway; 7 Intervention Centre Oslo University Hospital Oslo Norway

**Keywords:** eHealth, family, health care technology, infrastructure, palliative care, pediatric, pediatric palliative care, review, telemedicine

## Abstract

**Background:**

Families with children who need pediatric palliative care (PPC) often stay at home to preserve a sense of normalcy. However, families may experience challenges regarding communication and follow-up from health care professionals (HCPs). Health technology is suggested as a way to facilitate communication between families and HCPs, but no previous scoping review has mapped existing studies on health and communication technologies and infrastructures for supporting children who need PPC and their families.

**Objective:**

The objective of this scoping review was to systematically map the literature on health technologies and infrastructures to support communication in home-based PPC.

**Methods:**

We conducted a scoping review based on Arksey and O’Malley’s framework with a systematic search for relevant publications in the ASSIA, CINAHL, Embase, MEDLINE, PsycINFO, and Web of Science databases in November 2023, updated on August 28, 2025. Eligible publications comprised children (aged 0-18 years) with life-limiting or life-threating conditions requiring PPC and their families; HCPs, social care workers, or teachers caring for children in need of PPC and using any health technologies and infrastructures to support 2-way communication in home-based care; and literature published between January 1, 2018, and August 28, 2025, in Danish, English, Norwegian, or Swedish. Pairs of authors independently assessed eligibility and extracted data, which were summarized using a descriptive approach.

**Results:**

This review included 41 publications: 20 empirical papers, 6 protocol papers, 7 abstracts, 3 brief publications, 2 review papers, and 3 case publications. In 29.3% (12/41) of the publications, the researchers applied user-centered phased-design approaches to develop health technology for PPC. Children with cancer were most often studied in the publications. The most frequent delivery of health technology for communication in home-based PPC combined asynchronous and synchronous modes (19/41, 46.3%). Furthermore, the most frequent health technology apps for communication in home-based care were symptom monitoring apps (15/41, 36.6%), video technology (8/41, 19.5%), and health monitoring and video technology (3/41, 7.3%). Smartphones (14/41, 36.6%), internet and Wi-Fi (12/41, 29.3%), computers or laptops (9/41, 22%), and tablets (9/41, 22%) were the most frequently reported infrastructures.

**Conclusions:**

Children with cancer and their families are the most frequently reported users of health technology for communicating with HCPs in home-based PPC. However, research on children with diagnoses other than cancer and their families is limited. Combining asynchronous and synchronous modes is the most frequent way to deliver health technology, and children and their families often communicate with HCPs using symptom monitoring apps. Reports of health technology infrastructure for home-based PPC were insufficiently accounted for. Future studies should strive to include the voices of children in the development of health technology to align more closely with their needs.

**Trial Registration:**

Open Science Framework t9h4c; https://osf.io/t9h4c/

## Introduction

Children aged 0-18 years with life-limiting or life-threatening conditions or terminal illnesses could benefit from pediatric palliative care (PPC) [1]. Life-limiting conditions include children where a premature death is expected due to lack of a reasonable cure [2,3]. Life-threatening conditions include conditions such as congenital anomalies, cancer, and neurological conditions where there is a high probability of premature death, but where there remains a chance of survival into adulthood [2,3]. The proportion of children in need of PPC is increasing across the world [1]. PPC is defined as “the active total care of the child’s body, mind and spirit, and [this] also involves giving support to the family” [4]. PPC aims to optimize quality of life and care for these children and to address their wishes, choices, and needs and those of their families [1,5]. However, such children experience burdensome symptoms, concerns, and health outcomes that interact and occur during their illness trajectories [6,7]. PPC should be introduced at the time of diagnosis and provided throughout children’s lives [1,5,7].

Many families with children who need PPC tend to stay at home to preserve a sense of normalcy [2,8]. However, such families report a lack of access to out-of-hours support for addressing complex care needs. They also lack educational support and resources related to symptom management and their caregiving roles [9]; for example, how to manage symptoms to keep their children comfortable and free from pain and discomfort [10]. Similarly, families need to monitor their children’s symptoms, physical conditions, and well-being so that they can intervene when necessary [11]. Health care professionals (HCPs) emphasize that providing support for children’s needs can strengthen parents’ ability to manage their children’s pain and discomfort, thereby helping children and families articulate their experiences and describe the physical changes the children experience [12]. Families frequently need to contact hospitals for consultations or assistance, initiate contact with community health services, and plan and coordinate care [13,14]. However, they may feel powerless, helpless, and isolated [13].

The use of health technology for home-based PPC may be a way to enhance communication between home-based families and HCPs to support the needs of children and their families. We define health technology as information and communication technologies broadly used to enhance person-centered care, including telehealth, telemedicine, digital health, mobile health, and eHealth [15]. Health technology can benefit children and their families in home-based PPC by supporting them in communicating with HCPs, especially in discussing care goals and caregiver well-being [16]. A retrospective chart review revealed that children with cancer were usually seen in person, while children with neurologic diagnoses, medical technology dependence, and a higher number of complex chronic conditions were more often monitored using health technology. However, health outcomes, end-of-life quality metrics, and encounter-level quality indicators were similar across care delivery methods [17]. Another retrospective chart review suggested that PPC health technology, including telemedicine consultation, paralleled face-to-face consultation in terms of documented consultation components [18]. In an online survey, around 67% of children in need of PPC and their parents reported that they were willing to use health technology apps [19]. However, families and HCPs had concerns about health technology, with too many functions being confusing and potentially leading to technological overload [19]. HCPs may perceive health technology as useful for enhancing communication and maintaining relationships and as a time-efficient approach to ensuring that all relevant parties are updated and included in health-related discussions [20]. Furthermore, HCPs highlight the potential of one shared digital platform that can store and exchange pertinent health care information to enable HCPs to deliver timely and appropriate home-based PPC [15].

Several previous reviews have examined the use of health technology in home-based palliative care for adult patients [21-23]. One examined the use of home-based health technology for PPC but did not limit the included studies to children aged 0-18 years [24].

A previous scoping review explored the existing literature on the use of health technology for home-based PPC for children aged 0-21 years. The results suggest that health technology is acceptable for both families and HCPs [25]. A recent mixed methods review identified and reviewed the use of health technology for communication and to support home-based PPC [26]. The review found that health technology should be easy to use and effective in enhancing support and communication. However, this review did not systematically map the characteristics of health technologies and infrastructures for supporting communication in home-based PPC.

Health technology is frequently used for home-based PPC. PPC is provided throughout children’s lives [1,5,7], while adult palliative care is provided during a more limited period of the illness trajectory. Children also have different requirements when using a digital tool than adults. Consequently, evidence from adult home-based palliative care is not immediately transferable to the PPC context. Furthermore, no previous scoping review has mapped existing studies on health and communication technologies and infrastructures for supporting children in home-based PPC and their families. Such a review could provide important knowledge about existing technologies and infrastructures to help HCPs, care managers, and researchers implement health technology for home-based PPC. Facilitating the use of existing technologies rather than developing new technologies to support communication between families and HCPs would conserve resources and reduce costs. Consequently, in this scoping review, we aimed to systematically map the literature on health technologies and infrastructures for supporting communication in home-based PPC.

## Methods

### Study Design

We based this scoping review on the methodological framework of Arksey and O’Malley [27] and current methodological guidance [28,29]. Arksey and O’Malley’s framework consists of the following stages: identifying the research questions; identifying relevant studies; selecting studies; charting the data; and collating, summarizing, and reporting the results. Scoping reviews address broader topics and less specific research questions than systematic reviews and can include studies with different research designs and methods in addition to gray literature [27]. In line with the methodological guidance [27], we did not appraise the methodological quality of the included publications [30]. We reported the review according to the PRISMA-ScR (Preferred Reporting Items for Systematic Reviews and Meta-Analyses Extension for Scoping Reviews) checklist [30] (Multimedia Appendix 1). Deviations from the published protocol [31] are reported in Multimedia Appendix 2.

### Identifying the Research Questions

We formulated the following research questions: (1) What is known about existing health technologies and infrastructures for supporting communication in home-based PPC? (2) How can existing knowledge about health technologies and infrastructures inform future health technology development for home-based PPC?

### Identifying Relevant Studies Through Eligibility Criteria

The eligibility criteria are described in Textbox 1 using the population (P), concept (C), and context (C) framework [29], including the period, type of literature, and language.

**Description of eligibility criteria.**

**Inclusion criteria**

- Population: Children aged 0-18 years with life-limiting or life-threatening illnesses in need of pediatric palliative care (PPC) and their families, or health care professionals, social care workers, or teachers caring for children who need PPC.
- Concept: Health technologies and infrastructures for supporting any 2-way (asynchronous or synchronous) health communication between children or families and health care professionals, social care workers, or teachers; and health technologies and infrastructures for supporting communication between health care professionals, social care workers, or teachers regarding children receiving PPC.
- Context: Home-based care and children’s homes defined as institutions.
- Period: January 1, 2018, to August 28, 2025.
- Types of literature: Studies regardless of designs and methods, any type of reviews, conference abstracts, conference proceedings, study protocols, guidelines, position papers, discussion papers or theoretical papers, publications, PhD, letters, commentaries, or editorials.
- Language: Danish, English, Norwegian, or Swedish.

**Exclusion criteria**

- Population: Adults aged 18 years and older, children with chronic or long-term illnesses not defined as life-threatening or life-limiting, and survivors of cancer; families of children not receiving PPC; and health care professionals, social care workers, or teachers caring for children not in need of PPC.
- Concept: One-way health and communication technologies and infrastructures that do not facilitate asynchronous or synchronous communication with health care professionals, social care workers, or teachers; only telephone follow-up; and health technologies and infrastructures for supporting communication between health care professionals, social care workers, or teachers regarding children who are not in need of PPC.
- Context: Outside children’s homes.
- Period: Before January 1, 2018, and after August 28, 2025.
- Types of literature: Master theses.
- Language: All other languages.

We included studies regardless of study design and methods and gray literature to identify a broad range of relevant publications. We limited the literature to publications in Danish, English, Norwegian, or Swedish (the languages understood by our research team). We chose the period for the searches (2018-2025) to identify up-to-date, relevant health technologies and infrastructures that can inform future research, the development of health technology and services, education, and clinical practice.

### Information Sources

We performed a comprehensive search on November 27, 2023, of the ASSIA, CINAHL, Embase, MEDLINE, PsycINFO, and Web of Science databases to identify relevant literature. The database search was updated on August 28, 2025. The updated search comprised publications within 2023-2025, due to technical settings in the databases, only allowing to limit the search to whole years. Thus, the updated search will contain duplicates from the initial search, and the updated search is consequently reported as one search rather than specified to each database.

The search strategy was built in MEDLINE by 2 academic librarians (Elisabeth Karlsen and Ingjerd L Ødemark) with expertise in systematic searches for medical research databases in collaboration with the research team members SAS, EB, and HH. The search strategy was piloted by SAS and EB. Then, the search strategy was peer reviewed according to the Peer Review of Electronic Search Strategies guidelines [32] by a third librarian (Camilla Thorvik). The final search strategy was adopted for the remaining databases. The search strategy covered three elements: (1) palliative care, (2) children, and (3) health technology (Multimedia Appendix 3). We manually searched the reference lists of included studies to perform forward citation tracking using Google Scholar, and we also manually searched the *Journal of Medical Internet Research* and the *Journal of Telemedicine and Telecare*.

### Study Selection

We transferred the publications identified from the database searches to EndNote (Clarivate) to remove duplicates and then transferred them to Covidence (Veritas Health Innovation). Covidence is a systematic review web tool that ensures that the study selection process is blinded and that 2 researchers independently assess all publications. In total, all authors screened the publications in 2 steps. We screened titles and abstracts, uploaded full-text publications to Covidence, and assessed them against the eligibility criteria. In both steps, conflicts among the authors were resolved by SAS or HH.

### Charting the Data

We developed a standardized data charting form using Microsoft Word and included the following data items: author, year, country, type of literature, aim of study, sample, research design (when applicable), results, aim of health technology, development of health technology, users of health technology, health technology, follow-up service, mode of delivery, and infrastructure. SAS and HH piloted the data charting form using 4 publications. Based on the pilot and discussions within the research team, we collapsed some of the data items and restructured the data charting form to facilitate an overview of the extracted information. SAS and HH extracted data from the included publications, while AW, KR, and WC checked the data accuracy against the publications.

### Collating, Summarizing, and Reporting the Results

The first (SAS) and last authors (HH) summarized and organized the data according to the standardized data charting form using a quantitative descriptive approach [33]. Data in each column of the data charting form were grouped based on similarities and differences across the included publications. For instance, studies with comparable aims were grouped based on similarities, the different groups were categorized, and then, the number of studies in each group was counted and summarized, and percentages were calculated. Studies reporting on the use of comparable health technology such as monitoring apps or reporting on the same type of infrastructure were grouped together. Furthermore, for instance, the different users of health technology (ie, children, families, HCPs, or others) and the modes of delivery (ie, asynchronous, synchronous, and both delivery modes) were counted and summarized across the publications, and then, percentages were calculated. The coauthors provided feedback and agreed on the summarization and organization of the data.

## Results

### Overview

**Figure 1:**
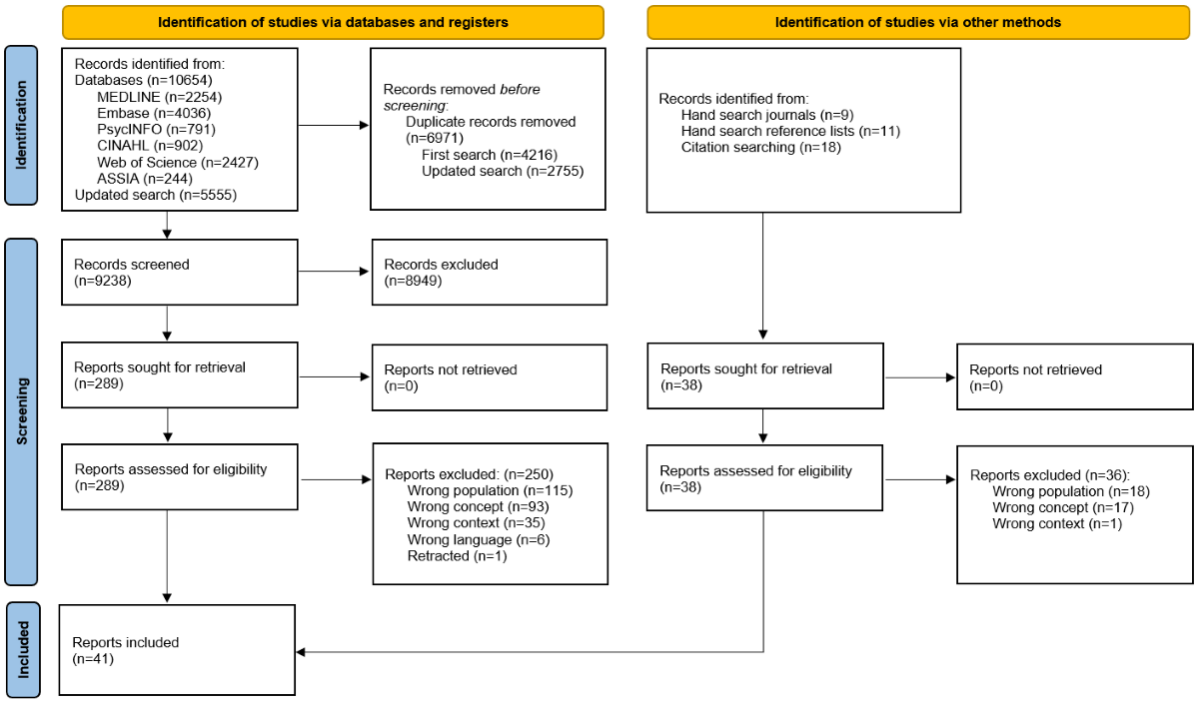
Flowchart of the selection process.

Through database searches, a total of 16,209 records were identified. After we deleted duplicates, we screened the titles and abstracts of 9238 records. We then assessed the full texts of 289 publications and included 39 publications. Two of these publications were identified in the updated database search. The manual searches yielded 2 eligible publications. The final sample consisted of 41 publications (Figure 1).

### Health Technology and Infrastructure

The characteristics of the health technologies and infrastructures reported in the included publications are described in Multimedia Appendix 4 [26,34-73].

#### The Aim of Health Technology

The publications described various aims of the health technology used for home-based PPC. In 34.1% (14/41) of the publications, the aims of the health technologies were to improve symptom management and reduce treatment burdens [34-47]. In 29.3% (12/41) of the publications, the aim of the health technologies was to support palliative care at home [26,48-58]; and in 12.2% (5/41) of the publications, the health technologies were designed to deliver psychosocial care and support psychosocial needs [59-63]. Furthermore, in 12.2% (5/41) of the publications, the aim of the health technologies was to support children and caregivers or parents [64-68]; in 9.8% (4/41), to improve clinical outcomes [69-72]; and in 2.4% (1/41), to improve communication and decision-making [73].

#### Development of Health Technology

In 29.3% (12/41) of the publications, the researchers used user-centered and phased-design approaches to develop health technologies for PPC [34,38,39,41,44,48,58,60-62,64,66]. Users such as children, adolescents, HCPs, and parents [34]; parents and professionals [41,44]; or parents [58,66] participated in the development processes. For instance, in one study, parents provided feedback at all stages of the intervention design and initial testing [61], while in another, adolescents with cancer provided feedback at all stages of app development and evaluation [38]. The health technology development processes included multiple phases; for instance, a needs assessment or analysis was conducted by interviewing children, caregivers, and HCPs or conducting a literature review [38,39,41,64,66]. The prototypes were refined and improved through usability testing (ie, think-aloud testing, beta testing by end users, and interviews with children and caregivers) [34,38,39,41,44,60-62,64].

In 4.9% (2/41) of the publications, the health technologies for home-based PPC were developed by an external web design company, while the educational information was provided by HCPs and a representative of a pain service center [46,47]. In 4.9% (2/41) of the publications, the health technologies or follow-up services were developed by HCPs or other professionals [49,71]. For instance, senior leadership (including clinical leadership), volunteer services leadership, project managers, and people with patient management system expertise were responsible for transitions to virtual hospices and the activities supporting such transitions. Furthermore, in one publication (1/41, 2.4%), the development of health technology was based on cognitive behavioral therapy [63], while in another publication (1/41, 2.4%), the development was based on an unreported theory and the experiences of children, families, and the research team [53]. Finally, in 3 publications (3/41, 7.3%), health technology for PPC was developed based on modifying a symptom self-monitoring system [40] or existing technology, such as a computer health evaluation system or EPIC [43,70]. However, in 46.3% (19/41) of the publications, the development of health technology and the inclusion of users in the development were not reported [26,35-37,43,45,50-52,54-56,59,65,67-69,72,73].

#### Users of Health Technology and Modes of Delivery

In 46.3% (19/41) of the publications, children, families, HCPs, and other professionals used health technology [26,35,36,40,41,44,46,47,49-51,53,57,63-65,67,72,73]; in 17.1% (7/41) of the publications, children and HCPs used health technology [34,37-39,42,43,45]; and in 36.5% (15/41) of the publications, families and HCPs used such technology [48,52,54-56,58-62,66,68-71].

The most frequent mode of delivery underpinning health technology for supporting communication in home-based PPC was a combined asynchronous and synchronous mode (19/41, 46.3%) [26,38-40,45-48,50,52,53,58,59,61,62,67,69,71,73] followed by asynchronous (12/41, 29.3%) [34,35,37,41-44,51,64-66,70] and synchronous (9/41, 22%) [36,49,54-57,63,68,72] modes.

#### Health Technologies and Follow-Up Services

The included publications described the diverse use of health technologies for communication between children or parents and HCPs and other professionals in home-based PPC.

The most frequent health technologies for communication regarding home-based PPC described in the publications (15/41, 36.6%) were symptom monitoring apps. In these publications, it was reported how children and parents used these apps to both assess and communicate children’s symptoms using child-adapted electronic patient-reported outcome measures or objective outcomes reported by the children or their parents [34,35,41-43,45-47,58] or how children self-reported pain or other symptoms using electronic diaries [37] or through gamification [38,39,44,64,65]. In some of these apps, if the reported symptoms were above predefined thresholds, HCPs received alerts and acted accordingly by contacting parents or adolescents to solve the problem [34,35,37-39,41-47,64,65]. Gamification was, for example, introduced to enable adolescents with cancer aged 12-18 years to act as law-enforcement officers or superheroes, scoring pain twice daily. If the adolescents reported pain, they received real-time pain assessments from the app and had to perform new pain assessments within 1 hour. However, if pain scores were above a certain level, nurses were alerted and contacted both the adolescents and medical teams to discuss and initiate interventions [38,39].

In several publications (8/41, 19.5%), video technology facilitated communication during videoconferences and telemedicine visits with children, families, and HCPs as well as provided remote outpatient clinics and music therapy [26,49,51,55-57,72,73]. During videoconferences, nurses participated in person from the children’s homes, while PPC physicians participated remotely from hospitals [51,55,56,73]. Nurses communicated with families about what to discuss with remote physicians, conducted physical examinations, and assessed the children to support physicians’ remote observations [51].

In a few publications (3/41, 7.3%), communication between parents and HCPs was facilitated by health monitoring and video technology*.* Children’s health was continuously remotely monitored, or parents provided data regarding the children’s health by entering the data. In addition, follow-ups consisted of video check-ups or weekly video visits with nurses or home-based HCPs [36,48,71]. Furthermore, in one publication, hospital HCPs could also send secure messages to family members and home-based HCPs [48]. In another publication, video technology was included in a web program for children with cancer aged 9-18 years and their families. The children received web-based training, coaching interviews, and counseling via video mobile calls, mobile messages, and children’s stories; progressive muscle relaxation and breathing exercises; and visualization interventions. The parents were offered video interviews with the coaches [67]. In one publication (1/41), children and other family members participated in an online chat group consisting of 6-8 members using a chat box. These sessions were led by a psychologist and a social worker [63].

In other publications (6/41, 14.6%), communication between parents and HCPs in home-based PC was facilitated using web-based technology [26,59-62,66,73]. Parents received interventions for psychosocial care and support through web programs, including modules that facilitated interactive self-directed sessions (ie, using a mix of video content and skills practice) [59-62] or interactive activities, such as videos and fotonovellas [66]. Parents received follow-up from telehealth guides, who had access to all the data entered by the parents. The publications did not report how such telehealth sessions were delivered [59-62]. In another publication (1/41, 2.4%), a counselor tracked the parents’ progress and provided follow-up if necessary [66].

In a few publications (3/41, 7.3%), electronic health record (EHR)–based systems facilitated communication regarding home-based PPC [40,52,70]. In one publication (1/41, 2.4%), the EHR system was used for the self-monitoring of symptoms, and children or parents assessed and reported symptoms. When symptoms exceeded a certain threshold, an email alert was sent to HCPs to initiate calls if necessary [40]. For instance, in another publication (1/41, 2.4%), families had access to portions of their children’s EHRs to view recorded information, schedule appointments, refill prescriptions, and ask HCPs questions [70]. Finally, in the third publication (1/41, 2.4%), families monitored and uploaded data and participated in video consultations with HCPs using EHR software [52].

#### Infrastructure

Around one-third of the publications accounted for health technology infrastructures, such as internet and Wi-Fi (12/41, 29.3%) [26,35,40,49,50,52,59,62,67,70,72,73], access to mobile data (2/41, 4.9%) [59,62], and an app that could be used when the internet was unavailable or inadequate (1/41, 2.4%) [44]. In addition, hotspots were used to reduce Wi-Fi barriers (2/41, 4.9%) [59,62].

Several types of devices, such as computers or laptops (9/41, 22%) [26,35,40,42,51,59,66,67,72], tablets (9/41, 22%) [26,40,42,47,48,52,66,71], or smartphones (14/41, 34.1%) [35,38-42,46,47,50,52,59,65-67,69], were used to access apps or web programs. A few publications (4/41, 9.8%) reported that parents could borrow smartphones or tablets for projects [38,42,52,62]. Publications (18/41, 43.9%) reported 14 different mobile apps [26,34,36,38,39,41,43-47,52,58,64,65,67,69,72,74] and 1 web app (1/41, 2.4%) [37]. One publication (1/41, 2.4%) reported that the app could be downloaded from Apple and Google Play Stores, while another (1/41, 2.4%) reported that there were 2 different versions of the app for children (based on the age of the children) and a family version [46].

Health technology infrastructures also included 12 different types of videoconferencing platforms, such as FaceTime, Zoom, Microsoft Teams, Near Me, and Attend Anywhere (9/41, 22%) [49,51-53,55,56,71-73]. Web cameras and headsets [26] or internet protocol cameras with pan-tilt-zoom [50] were used. Furthermore, in one publication (1/41, 2.4%), a chat box was used to facilitate written communication [63]. Some publications (3/41, 7.3%) reported the use of website infrastructure, such as Bespoke and MyQuality [26,67,73], web-based patient portals (1/41, 2.4%) [42], or web programs (2/41, 4.9%) [61,62]. Other publications (4/41, 9.8%) reported the use of EHR systems such as EPIC, My Chart, and Symon-SAYS [40,52,53,70].

In total, 3 publications (3/41, 7.3%) reported 3 different systems for monitoring children’s health conditions, including the Cloud DX Connected Health System (Google Inc) and Medlinecare 2.1 (Medical Online Technology SL), which monitor children’s conditions in real time at home from pediatric intensive care units. Electronic monitoring devices for such monitoring could include a pulse oximeter, scale, or tele-auscultation [48,50,71].

Infrastructure-related aspects mentioned in the publications (4/41, 9.8%) related to the content of technology, such as web-based resources, including video content that appealed to children of different ages and parents, hands-on web-based activities, graphic publications, worksheet tools, and fotonovellas [40,61,62,66,67].

The reported infrastructures also facilitated data security and privacy. Publications reported the use of secure apps, platforms, portals, or servers (7/41, 17.1%) [38,49,51,62,63,71,72], the storage of app data on secure clouds (1/41, 2.4%) [34], and the use of 2-factor authentication (2/41, 4.9%) [46,47]. In addition, in one publication, data entered into a website were controlled by patients or caregivers who could grant access to HCPs [73], and in another publication (1/41 2.4%), data were not linked to the children’s EHRs [34]. In total, 3 (7.3%) publications did not provide information regarding infrastructure [54,59,68].

### Description of the Included Literature

The final sample consisted of 41 publications: 20 empirical papers, 6 protocol papers, 7 abstracts, 3 brief publications, 2 review papers, and 3 case publications. The characteristics of the included publications are described in Multimedia Appendix 5 [26,34-73].

#### Empirical Papers

The empirical papers reported on studies conducted in the United States (9/20, 45%), Canada (2/20, 10%), Iran (2/20, 10%), the Netherlands (2/20, 10%), Austria (2/20, 10%), China (1/20, 5%), Indonesia (1/20, 5%), and Spain (1/20, 5%). The sample sizes reported in the empirical papers ranged from 12 to 621 participants.

The empirical papers included children (7/20, 35%) [39,42,47,52,56-58]; children and families (3/20, 15%) [50,65,71]; children, families, HCPs, and other professionals (3/20, 15%) [41,55,64]; children and HCPs (1/20, 5%) [46]; families (5/20, 25%) [44,60,61,66,70]; and families and HCPs (1/20, 5%) [48].

In 70% (14/20) of the empirical papers, the children had been diagnosed with cancer, or the families had children with cancer. In total, 13 of 20 (65%) empirical studies were based on quantitative methods [42,47,50,52,55,57,58,61,65,66,70,71], 5 of 20 (25%) on mixed or methods [41,44,45,48,60], and 3 of 20 (15%) on qualitative methods [39,64,71]. A total of 12 of 20 (60%) empirical studies assessed the feasibility, usability, usefulness, acceptability, or benefits of health technologies [36,39,42,46,48,50,55,57,58,61,65,71], while 4 of 20 (20%) aimed to develop health technologies for PPC [41,44,60,64].

#### Protocol Papers

The protocol papers described studies planned in the United States (3/6, 50%), Australia (1/6, 17%), Canada (1/6, 17%), and Turkey (1/6, 17%). These papers aimed to include children (3/6, 50%), children and families (1/6, 17%), children and HCPs (1/6, 17%), or families only (1/6, 17%). Most of these studies (5/6, 83%) targeted children or families of children with cancer. In total, 5 of 6 (83%) protocol papers described plans for randomized controlled trial designs [38,40,53,62,67], and 1 of 6 (17%) described a mixed methods approach [34].

#### Review Papers

The 2 review papers were conducted in Norway (1/2, 50%) and the United States (1/2, 50%). One paper was a convergent systematic mixed methods review that covered 7 empirical papers [26], while the other used no systematic search and study selection method and included 112 references [35].

#### Brief Publications

The 3 brief publications were from Austria (1/3, 33%), the United Kingdom (1/3, 33%), and the United States (1/3, 33%). These papers included children (1/3, 33%) [36], nurses (1/3, 33%) [54], and empirical studies (1/3, 33%) [73]. One paper included 152 children with cancer [36], another included 15 nurses [54], and the third included 3 empirical studies (prospective longitudinal studies) [73]. The brief report papers used retrospective cost analysis [36], qualitative design (1/3, 33%) [54], and a systematic review and narrative synthesis (1/3, 33%) [73].

#### Case Publications

The 3 case publications were from Australia (1/3, 33%), Austria (1/3, 33%), and the United Kingdom (1/3, 33%). One case report included a pediatric hospice provider (1/3, 33%) [49], another included an anecdotal case about a 10-year-old child with cancer (1/3, 33%) [43], and the third included 3 children (1/3, 33%) [72].

#### Conference Abstracts

The 7 identified conference abstracts were from the United States (3/7, 43%), Canada (1/7, 14%), Colombia (1/7, 14%), India (1/7, 14%), and the Netherlands (1/7, 14%). The abstracts included samples of children (3/7, 43%); children, families, and HCPs (2/7, 29%); families (1/7, 14%); and families and HCPs (1/7, 14%). In 50% (3/6) of the abstracts, the samples comprised children with cancer or family members of children with cancer. In total, 1 abstract referred to a pilot test [59], 1 to a randomized controlled trial [37], 1 to a cross-sectional study [68], and 1 to a mixed methods study [45]. A total of 2 abstracts did not report methods [63,69], while 1 reported no methods [51].

## Discussion

### Principal Findings

The aim of this scoping review was to systematically map the literature on health technologies and infrastructures for supporting communication in home-based PPC.

We identified and summarized numerous technologies, features, and their associated infrastructures. Overall, in a limited number of publications, the users (ie, children and caregivers) participated in the technology development process for home-based PPC, and children with cancer and their caregivers were the most frequently reported users. The most frequent delivery mode for such technology was via combined asynchronous and synchronous modes. Apps for symptom monitoring were the most frequent health technology apps for communication regarding home-based PPC. However, details of health technology infrastructures for home-based PPC were lacking or insufficiently accounted for in the publications.

Our review revealed that only 29.3% of the publications included users, such as children, caregivers, and HCPs, in the development of health technology and follow-up services. Furthermore, around 46% of the publications provided no information about the development processes or the participation of users. Consequently, we may assume that users were not included in the development in these cases. This omission was unsurprising, as another scoping review claimed that children were rarely included in research before and after interventions [75]. However, users may have been included in the development process, but this information may have been omitted from the papers due to the word limitation, or the researcher may be unsure what to report regarding the inclusion of users. It is imperative for users to participate in developing and tailoring health technology to align more closely with patient needs [21] and to avoid posing additional strain and burden on these families causing harm. Furthermore, adults (ie, caregivers and HCPs) may not fully understand what children experience or understand and what their needs might be [76,77]. Therefore, including children in the development of health technology is crucial for designing effective and useful communication systems. Services for children in need of PPC must be tailored to their ages, developmental capacities, and preferences [78]. Future studies should strive to include children in the development of health technology and follow-up services. However, such inclusion could be challenging due to children’s health conditions. Developing personas based on, for instance, interview data about the needs of children in PPC may be one approach to include children’s voices in the development process. Personas are concrete, specific, and fictitious representations of end users and represent end users with common behavioral features that may facilitate designers’ ability to develop usable and useful designs (ie, of health technology) [79] and engage users in expressing their needs [80]. Furthermore, future studies should develop guidelines for reporting user participation, especially when children are included in the development of health technology. This could enhance the transparency and the reliability of such development.

We found that the most frequently reported mode of delivery of health technology for communication combined asynchronous and synchronous modes. This could enable children or caregivers to report symptoms and needs at home, while HCPs are remotely available for communication, support, and interventions when needed. This approach may enhance caregivers’ perceptions of normalcy, their ability to maintain a normal family life, and their feelings of being supported at home [2,8]. Furthermore, caregivers may need easily accessible long-term support from HCPs to manage difficult situations in the future, especially for patients discharged from the hospital to home-based care [81]. Bergstraesser [82] emphasized the importance of care delivery that is flexible, individualized, and considers the individual needs of children and their caregivers. Consequently, future studies should be aware that combining asynchronous and synchronous modes of delivery may facilitate children’s and caregivers’ access to and support from HCPs.

In around 17% (7/41) of the included publications, children used health technology autonomously, while in around 46% (19/41) of the publications, children used technology together with their caregivers to communicate with HCPs. The Convention on the Rights of the Child advocates for promoting children’s interests and views [76]. Health technology could facilitate children’s ability to express their needs to both their caregivers and HCPs. Children, especially adolescents, may appreciate direct communication with HCPs rather than communicating through their parents [81], and similarly, HCPs value the opportunity to communicate directly with children [26]. In most of the included publications reporting on children actively participating in their care using health technology, the children had cancer diagnoses. However, children with diagnoses other than cancer may have other PPC needs, and some may have difficulty expressing themselves verbally and may communicate using eye movements [78,83]. Furthermore, children with diagnoses other than cancer may receive PPC from multiple sites, and their caregivers may lack a support system and feel isolated [78]. Health technology could mitigate the logistical issues associated with in-person consultations for children with complex medical needs, prevent interruptions to the children’s routines or medical care, and ease the complications of moving children who are medically vulnerable [84]. Future studies should develop or tailor health technology for children with primary diagnoses other than cancer and their caregivers to align more closely with their needs and facilitate communication with HCPs in the broader context of home-based PPC.

We found that the most frequently used health technology apps for communication in home-based PPC were symptom-monitoring apps. This attention to symptom monitoring is not surprising, as children in PPC, regardless of their diagnoses, often experience burdensome symptoms during their illness trajectories [7]. The aim of PPC is to prevent and alleviate burdensome symptoms and improve quality of life [1], and symptom assessment is a prerequisite for optimal symptom management. Apps that allow children or caregivers to report children’s symptoms to HCPs in real time can facilitate and improve HCPs’ understanding of children’s conditions [35]. In several of the included publications, children used apps including patient-reported outcomes to communicate their symptoms to HCPs. Children’s voices are crucial, since self-reporting of symptoms is the gold standard for assessing symptoms, given that caregivers and HCPs often underreport the number and severity of symptoms children experience [35]. Patient-reported outcome data may improve care delivery by shaping children’s and HCPs’ expectations, facilitating communication about important issues, and enhancing shared decision-making, which might facilitate interventions to address children’s specific needs [35]. HCPs need to tailor care and symptom management to children according to their cognitive, emotional, social, and physical stages of development [1]. The use of existing symptom monitoring apps rather than developing new ones may conserve resources and enhance sustainability, and may be transferable to children with complex needs such as diabetes. However, as PPC includes a heterogeneous group of children with diverse and complex needs as well as various abilities to use such apps, these apps need to be tailored to both the conditions, the needs of the individual child, and the services there are to function within. Notably, only a few of the publications used gamification to allow children to report their symptoms. This absence is not surprising since gamification has been applied to a limited extent in PPC [85]. Gamification may support children’s creativity and provide them with choices; children may enjoy the rewards, incentives, and personalized aspects of such apps, which may also ease symptom reporting and help in specifying symptom locations [86]. However, a recent scoping review on gamification in mobile apps for children with disabilities demonstrated potential benefits across different populations but highlighted mixed results due to gamification’s impact on the health, health behavior outcomes, and health development of children with disabilities [87]. Future studies should investigate whether and how gamification could be used in home-based PPC to facilitate communication between children, caregivers, and HCPs. Studies should also investigate the possible side effects of gamification.

Our findings suggest that the internet, Wi-Fi, and mobile data are common infrastructures needed to use health technologies for home-based PPC. Even though such infrastructures were only reported in around 36% of the publications, we assumed that they were prerequisites for the technologies reported in all the publications. Information about infrastructure is essential for policymakers, health care managers, and HCPs who want to implement health technology in home-based PPC by providing information about what is needed to secure the implementation of such services and to assess resource use and cost as well as the need for training for HCPs. Dependence on access to the internet creates a new vulnerability for communication in home-based PPC and may impose an additional burden on children and parents as well as HCPs. A scoping review found that unreliable technology and connectivity issues may undermine HCPs’ confidence in using health technology, and that HCPs may feel professionally and personally responsible if health technology solutions fail [23]. Only a few publications reported measures to mitigate such risk, such as an app that could be used when the internet did not function properly [44] and hotspots to reduce Wi-Fi barriers [59,62]. Mobile devices (ie, smartphones, tablets, and laptops) could also create challenges for caregivers who cannot afford such devices. The costs involved could increase health inequity and the digital divide. For instance, purchasing technology or an expensive internet plan may be a heavy financial burden that can limit technology access for families with several children. One potential solution for enhancing technology access for families in home-based PPC could be that hospitals or home care services lend out devices such as smartphones or tablets to families. Furthermore, few technology resources and low technological literacy may hinder people’s ability to engage in necessary health care activities, such as video consultations [74].

The included publications mainly accounted for the type, feature, and content of the health technology and follow-up from HCPs, while the infrastructure needed to use health technology was insufficiently reported or lacking. Furthermore, even though privacy and data security are prerequisites for using health technology in home-based PPC, only a few publications have addressed these issues. Privacy and confidentiality may pose important legal and ethical challenges for the use of health technology, especially during video consultations. Furthermore, HCPs using health technology in home-based palliative care have access to large portions of patients’ daily life data, including a great deal of sensitive information. These sensitive data must be protected against unauthorized access and misuse [88]. An overview of the infrastructure may be essential to tailor or implement existing health technology in home-based PPC to save costs and resources and enhance sustainability. Consequently, future studies should develop a checklist for reporting health technology infrastructure and health economic evaluations.

### Strengths and Limitations

A strength of our review was that we registered the review protocol a priori and used an acknowledged methodological framework to guide our review. We developed the search strategy in collaboration with experienced research librarians, and independent pairs of researchers conducted the study selection and data extraction. The inclusion of studies was limited to 2018-2025, which enabled us to identify and map up-to-date, relevant health technologies for home-based PPC. However, relevant health technologies may have been reported in studies published before 2018, which we were not able to identify. Another strength was that we searched for and included diverse literature to enhance the mapping of the existing technology for home-based PPC. However, the conference abstracts provided only limited descriptions of health technologies and infrastructures. A limitation is that we may not have identified all the relevant search terms for PPC and health technology. Our inclusion criteria restricted the review to only publications in the English and Scandinavian languages. We did not search specific databases for gray literature, but the type of literature included in our review is indexed in the chosen databases. Consequently, there may be publications that we did not identify. Furthermore, our findings may have some transferability to children with complex care needs, but it is different for children to live with a life-limiting or life-threatening condition than a chronic illness such as diabetes. However, regarding transferability, HCPs and researchers must assess whether our findings are relevant for their context.

### Conclusions

Based on the included publications, children with cancer and their families are the primary users of health technology to communicate with HCPs in home-based PPC, although there is limited use among children with diagnoses other than cancer and their families. Combined asynchronous and synchronous modes are the most frequent modes of health technology delivery. Furthermore, children and their families often communicate with HCPs using symptom monitoring apps. The reporting of health technology infrastructures for home-based PPC is lacking or insufficiently accounted for.

Future studies should strive to include the voices of children, especially children with diagnoses other than cancer, to align health technology more closely with their needs. Studies should explore and evaluate whether gamification is feasible for children who require PPC and potentially develop such health technology for home-based PPC. Reporting guidelines for the inclusion of users in the development of health technology, especially children, as well as health technology infrastructure would be beneficial for supporting clinicians and researchers in gaining an overview of the infrastructure needed to implement health technology and follow-up services. This may conserve resources, reduce costs, and prevent research waste.

## Appendix

Multimedia Appendix 1 PRISMA-ScR checklist.

Multimedia Appendix 2 Deviations from the published protocol.

Multimedia Appendix 3 Search strategy in all the 6 databases.

Multimedia Appendix 4 Description of health technologies and infrastructures.

Multimedia Appendix 5 Characteristics of the included publications.

## Abbreviations

EHR: electronic health record
HCP: health care professional
PPC: pediatric palliative care
PRISMA-ScR: Preferred Reporting Items for Systematic Reviews and Meta-Analyses Extension for Scoping Reviews
