# Supplementary material for: Health Technologies and Infrastructures for Supporting Home-Based Pediatric Palliative Care: Scoping Review
Source: J Med Internet Res. 2025 Dec 8;27:e70687. doi: 10.2196/70687 (PMC12723365; doi:10.2196/70687)
Supplement: Multimedia Appendix 2 [file jmir_v27i1e70687_app2.docx]

| **Approach described in the protocol** | **Description of the**  **deviation from protocol** |
| --- | --- |
| We will also hand search study registers such as clinicaltrials.gov and Cochrane Central Register of controlled trials. | Studies registered in these study registers were identified through the database Medline. Consequently, it was not necessary to perform additional searches in these registers. |
| Inclusion criteria, type of literature: Master’s thesis. | Exclude master’s thesis as master’s thesis is not indexed in the databases we searched. |
| The research team developed a data charting form in Covidence to extract data from the included literature. | The data charting form was instead developed in Word document as facilitated better overview of the data charting than in Covidence. |
| The research team will develop a data charting form in Covidence to extract data from the included literature which. | We developed a standardized data charting form using Microsoft Word document. |
| The data charting form may include the following data items: Author, year, country; type of literature; aim; sample; research design when applicable; health technology and service; infrastructure; findings relevant for research question 1. | We developed a standardized data charting form using Microsoft Word document that included the following data items: Author, year, country; type of literature; aim of study; sample; research design when applicable; results; aim of health technology, development of health technology, users of health technology; health technology, follow-up service, mode of delivery; and infrastructure. |
| The research team will develop a data charting form in Covidence to extract data from the included literature which will be piloted by SAS and HH on 2-5 publications. Three pairs of researchers will extract data; one will extract data, while the other will check data accuracy  against the publication. | SAS and HH extracted data from the included publications, while AW, KR and WC checked data accuracy against the publications. |
