# Supplementary material for: Health Technologies and Infrastructures for Supporting Home-Based Pediatric Palliative Care: Scoping Review
Source: J Med Internet Res. 2025 Dec 8;27:e70687. doi: 10.2196/70687 (PMC12723365; doi:10.2196/70687)
Supplement: Multimedia Appendix 3 [file jmir_v27i1e70687_app3.docx]

Database: Medline via Ovid Ovid MEDLINE(R) and Epub Ahead of Print, In-Process, In-Data-Review & Other Non-Indexed Citations and Daily <1946 to November 22, 2023>

| **#** | **Searches** |
| --- | --- |
| 1 | Palliative Care/ or "Hospice and Palliative Care Nursing"/ or Terminal Care/ or Palliative Medicine/ or exp Advance Care Planning/ or Terminally ill/ or Hospice care/ |
| 2 | palliat*.tw,kw,kf. |
| 3 | ((terminal* or end stage* or endstage* or advanced stage* or late stage*) adj3 (disease* or ill* or care* or caring or treatment* or period* or nurs* or patient*)).tw,kw,kf. |
| 4 | ((incurabl* or irreversibl*) adj ill*).tw,kw,kf. |
| 5 | (eol or "end of life" or dying).tw,kw,kf. |
| 6 | (advance*1 adj3 (plan*1 or planning or directive*)).tw,kw,kf. |
| 7 | hospice*.tw,kw,kf. |
| 8 | ((life limit* or life threatening) adj3 (disease* or condition* or illness*)).tw,kw,kf. |
| 9 | (advance* adj (disease* or illness*)).tw,kw,kf. |
| 10 | Neoplasms/ or Cerebral Palsy/ or Brain Injuries/ or Spinal Cord Injuries/ |
| 11 | (cancer* or neoplasm* or cerebral pals* or brain injur* or spinal cord injur*).tw,kw,kf. |
| 12 | or/1-11 |
| 13 | telemedicine/ or telepathology/ or teleradiology/ or telerehabilitation/ |
| 14 | remote sensing technology/ |
| 15 | videoconferencing/ or webcasts as topic/ or webcast/ |
| 16 | Telecommunications/ or Wireless Technology/ |
| 17 | telephone/ or answering services/ or cell phone/ or smartphone/ or text messaging/ |
| 18 | Mobile Applications/ or computers/ or microcomputers/ or computers, handheld/ or minicomputers/ or User-Computer Interface/ or Computer assisted instruction/ |
| 19 | Internet-Based Intervention/ |
| 20 | (((wearable or wireless or portable) adj2 (technolog* or electronic* or device*)) or (digital adj2 medicine) or (technolog* adj2 (remote or health)) or (remote adj2 care) or ((mobile or internet or electronic* or robot* or remote or virtual or wireless) adj2 (consultation* or app* or device* or rehab* or communicat* or team*))).tw,kw,kf. |
| 21 | (telecommunicat* or tele communicat* or teleconferenc* or tele conferenc* or app or apps or mobile based or short message* service* or sms or textmessag* or text messag* or texting or videoconferenc* or video conferenc* or webconferenc* or web conferenc* or webcast* or web cast* or webinar* or web based or web camera* or web application*).tw,kw,kf. |
| 22 | (((electronic or mobile or digital) adj health) or ((information or communicat*) adj technolog*) or "application of technolog*").tw,kw,kf. |
| 23 | (smartphone* or smart phone* or cellphone* or cell phone* or mobilephone* or mobile phone* or personal digital assistant* or palmpilot* or palm pilot* or smarthome* or smart home* or touchscreen* or touch screen* or high tech* or hightech*).tw,kw,kf. |
| 24 | (telemedicin* or tele medicin* or telehealth* or tele health* or telecare* or tele care* or telecari* or tele cari* or emedic* or e medic* or ehealth* or e health* or mhealth* or m health* or ehomecare* or e homecare* or e home care* or telehomecare or tele homecare or tele home or telenurs* or tele nurs* or teletherap* or tele therap* or telerehab* or tele rehab* or erehab* or e rehab* or teleconsultation* or tele consultation* or videoconsultation* or video consultation* or telemonitor* or tele monitor* or connected car*).tw,kw,kf. |
| 25 | ((internet based intervention* or (digital or online)) adj2 intervention*).tw,kw,kf. |
| 26 | or/13-25 |
| 27 | 12 and 26 |
| 28 | adolescent/ or puberty/ or child/ or child, preschool/ or infant/ or infant, newborn/ or infant, large for gestational age/ or infant, low birth weight/ or infant, small for gestational age/ or infant, very low birth weight/ or infant, extremely low birth weight/ or infant, postmature/ or infant, premature/ or infant, extremely premature/ |
| 29 | Pediatrics/ |
| 30 | Young Adult/ |
| 31 | (pediatric* or paediatric* or peadiatric* or neonatal* or neo natal* or neonate* or newborn* or new born* or infant* or baby or babies or toddler* or child* or kid or kids or girl or girls or boy or boys or minors or underage* or under age* or teen* or youth* or youngster* or adolescent* or adolescence or preadoles* or pre adoles* or juvenil* or puber* or pubescen* or pre puber* or prepuber* or prepubescen* or pre pubescen* or schoolchild* or preschool* or (young adj2 (adult* or man or men or woman or women or person* or people))).tw,kw,kf. |
| 32 | or/28-31 |
| 33 | 27 and 32 |
| 34 | limit 33 to yr="2018 -Current" |

Database: Embase

| **#** | **Searches** |
| --- | --- |
| 1 | cancer palliative therapy/ or palliative therapy/ or palliative chemotherapy/ |
| 2 | palliative nursing/ |
| 3 | terminal care/ or advance care planning/ or hospice care/ |
| 4 | terminally ill patient/ or hospice patient/ |
| 5 | palliat*.tw,kw,kf. |
| 6 | ((terminal* or end stage* or endstage* or advanced stage* or late stage*) adj3 (disease* or ill* or care* or caring or treatment* or period* or nurs* or patient*)).tw,kw,kf. |
| 7 | ((incurabl* or irreversibl*) adj ill*).tw,kw,kf. |
| 8 | (eol or "end of life" or dying).tw,kw,kf. |
| 9 | (advance*1 adj3 (plan*1 or planning or directive*)).tw,kw,kf. |
| 10 | hospice*.tw,kw,kf. |
| 11 | ((life limit* or life threatening) adj3 (disease* or condition* or illness*)).tw,kw,kf. |
| 12 | (advance* adj (disease* or illness*)).tw,kw,kf. |
| 13 | Neoplasms/ or Cerebral Palsy/ or Brain Injuries/ or Spinal Cord Injuries/ |
| 14 | (cancer* or neoplasm* or cerebral pals* or brain injur* or spinal cord injur*).tw,kw,kf. |
| 15 | or/1-14 |
| 16 | teleconsultation/ or electronic consultation/ |
| 17 | telemedicine robot/ or medical robot/ |
| 18 | telehealth/ or telecare/ or telenursing/ |
| 19 | telemedicine/ or telecardiology/ or teledentistry/ or teledermatology/ or telediagnosis/ or telemonitoring/ or telenephrology/ or teleneurology/ or telepathology/ or telepharmacy/ or telepsychiatry/ or telepsychology/ or teleradiology/ or teleradiotherapy/ or telerehabilitation/ or telesurgery/ or teletherapy/ or video consultation/ |
| 20 | remote sensing/ |
| 21 | telecommunication/ or videoconferencing/ or wireless communication/ or teleconference/ |
| 22 | webcast/ |
| 23 | mobile application/ or mobile health application/ |
| 24 | telephone/ or mobile phone/ or smartphone/ or computer/ or microcomputer/ or personal computer/ or personal digital assistant/ or computer interface/ or human machine interface/ or computer network/ |
| 25 | web-based intervention/ |
| 26 | (((wearable or wireless or portable) adj2 (technolog* or electronic* or device*)) or (digital adj2 medicine) or (technolog* adj2 (remote or health)) or (remote adj2 care) or ((mobile or internet or electronic* or robot* or remote or virtual or wireless) adj2 (consultation* or app* or device* or rehab* or communicat* or team*))).tw,kw,kf. |
| 27 | (telecommunicat* or tele communicat* or teleconferenc* or tele conferenc* or app or apps or mobile based or short message* service* or sms or textmessag* or text messag* or texting or videoconferenc* or video conferenc* or webconferenc* or web conferenc* or webcast* or web cast* or webinar* or web based or web camera* or web application*).tw,kw,kf. |
| 28 | (((electronic or mobile or digital) adj health) or ((information or communicat*) adj technolog*) or "application of technolog*").tw,kw,kf. |
| 29 | (smartphone* or smart phone* or cellphone* or cell phone* or mobilephone* or mobile phone* or personal digital assistant* or palmpilot* or palm pilot* or smarthome* or smart home* or touchscreen* or touch screen* or high tech* or hightech*).tw,kw,kf. |
| 30 | (telemedicin* or tele medicin* or telehealth* or tele health* or telecare* or tele care* or telecari* or tele cari* or emedic* or e medic* or ehealth* or e health* or mhealth* or m health* or ehomecare* or e homecare* or e home care* or telehomecare or tele homecare or tele home or telenurs* or tele nurs* or teletherap* or tele therap* or telerehab* or tele rehab* or erehab* or e rehab* or teleconsultation* or tele consultation* or videoconsultation* or video consultation* or telemonitor* or tele monitor* or connected car*).tw,kw,kf. |
| 31 | ((internet based intervention* or (digital or online)) adj2 intervention*).tw,kw,kf. |
| 32 | or/16-31 |
| 33 | 15 and 32 |
| 34 | adolescent/ or puberty/ or child/ or preschool child/ or infant/ or newborn/ or small for date infant/ or prematurity/ or pediatrics/ or school child/ or young adult/ or toddler/ |
| 35 | (pediatric* or paediatric* or peadiatric* or neonatal* or neo natal* or neonate* or newborn* or new born* or infant* or baby or babies or toddler* or child* or kid or kids or girl or girls or boy or boys or minors or underage* or under age* or teen* or youth* or youngster* or adolescent* or adolescence or preadoles* or pre adoles* or juvenil* or puber* or pubescen* or pre puber* or prepuber* or prepubescen* or pre pubescen* or schoolchild* or preschool* or (young adj2 (adult* or man or men or woman or women or person* or people))).tw,kw,kf. |
| 36 | or/34-35 |
| 37 | 33 and 36 |
| 38 | limit 37 to yr="2018 -Current" |

Database: **APA PsycInfo**1806 to November Week 2 2023

| **#** | **Searches** |
| --- | --- |
| 1 | palliative care/ or terminally ill patients/ or terminal cancer/ or "death and dying"/ |
| 2 | hospice/ or advance directives/ |
| 3 | palliat*.tw. |
| 4 | ((terminal* or end stage* or endstage* or advanced stage* or late stage*) adj3 (disease* or ill* or care* or caring or treatment* or period* or nurs* or patient*)).tw. |
| 5 | ((incurabl* or irreversibl*) adj ill*).tw. |
| 6 | (eol or "end of life" or dying).tw. |
| 7 | (advance*1 adj3 (plan*1 or planning or directive*)).tw. |
| 8 | hospice*.tw. |
| 9 | ((life limit* or life threatening) adj3 (disease* or condition* or illness*)).tw. |
| 10 | (advance* adj (disease* or illness*)).tw. |
| 11 | neoplasms/ |
| 12 | cerebral palsy/ |
| 13 | brain injuries/ |
| 14 | spinal cord injuries/ |
| 15 | (cancer* or neoplasm* or cerebral pals* or brain injur* or spinal cord injur*).tw. |
| 16 | or/1-15 |
| 17 | telecommunications media/ or communication systems/ |
| 18 | electronic health services/ or digital interventions/ or mobile health/ or wearable devices/ |
| 19 | telemedicine/ or online therapy/ or teleconsultation/ or telepsychiatry/ or telepsychology/ or telerehabilitation/ |
| 20 | teleconferencing/ or videoconferencing/ or groupware/ or telecommuting/ or virtual teams/ |
| 21 | digital technology/ or sensor technology/ or streaming technology/ or touchscreen technology/ |
| 22 | computer applications/ or mobile applications/ or mobile technology/ |
| 23 | computer assisted therapy/ |
| 24 | electronic communication/ or text messaging/ |
| 25 | computer mediated communication/ |
| 26 | wireless technologies/ |
| 27 | computer usage/ or computers/ |
| 28 | microcomputers/ |
| 29 | mobile devices/ or tablet computers/ |
| 30 | mobile phones/ or mobile devices/ or telephone systems/ or smartphones/ or cellular phones/ |
| 31 | (((wearable or wireless or portable) adj2 (technolog* or electronic* or device*)) or (digital adj2 medicine) or (technolog* adj2 (remote or health)) or (remote adj2 care) or ((mobile or internet or electronic* or robot* or remote or virtual or wireless) adj2 (consultation* or app* or device* or rehab* or communicat* or team*))).tw. |
| 32 | (telecommunicat* or tele communicat* or teleconferenc* or tele conferenc* or app or apps or mobile based or short message* service* or sms or textmessag* or text messag* or texting or videoconferenc* or video conferenc* or webconferenc* or web conferenc* or webcast* or web cast* or webinar* or web based or web camera* or web application*).tw. |
| 33 | (((electronic or mobile or digital) adj health) or ((information or communicat*) adj technolog*) or "application of technolog*").tw. |
| 34 | (smartphone* or smart phone* or cellphone* or cell phone* or mobilephone* or mobile phone* or personal digital assistant* or palmpilot* or palm pilot* or smarthome* or smart home* or touchscreen* or touch screen* or high tech* or hightech*).tw. |
| 35 | (telemedicin* or tele medicin* or telehealth* or tele health* or telecare* or tele care* or telecari* or tele cari* or emedic* or e medic* or ehealth* or e health* or mhealth* or m health* or ehomecare* or e homecare* or e home care* or telehomecare or tele homecare or tele home or telenurs* or tele nurs* or teletherap* or tele therap* or telerehab* or tele rehab* or erehab* or e rehab* or teleconsultation* or tele consultation* or videoconsultation* or video consultation* or telemonitor* or tele monitor* or connected car*).tw. |
| 36 | ((internet based intervention* or (digital or online)) adj2 intervention*).tw. |
| 37 | or/17-36 |
| 38 | 16 and 37 |
| 39 | ("100" or "120" or "140" or "160" or "180" or "200" or "320").ag. |
| 40 | child care/ or early adolescence/ or puberty/ or Child health/ or Pediatrics/ |
| 41 | (pediatric* or paediatric* or peadiatric* or neonatal* or neo natal* or neonate* or newborn* or new born* or infant* or baby or babies or toddler* or child* or kid or kids or girl or girls or boy or boys or minors or underage* or under age* or teen* or youth* or youngster* or adolescent* or adolescence or preadoles* or pre adoles* or juvenil* or puber* or pubescen* or pre puber* or prepuber* or prepubescen* or pre pubescen* or schoolchild* or preschool* or (young adj2 (adult* or man or men or woman or women or person* or people))).tw. |
| 42 | or/39-41 |
| 43 | 38 and 42 |
| 44 | limit 43 to yr="2018 -Current" |

Database: Cinahl EbscoHost

| **#** | **Query** |
| --- | --- |
| S1 | (MH "Palliative Medicine") |
| S2 | (MH "Palliative Care") |
| S3 | (MH "Hospice Care") OR (MH "Hospice Nursing") |
| S4 | (MH "Advance Care Planning") |
| S5 | (MH "Terminally Ill Patients") OR (MH "Terminal Care") |
| S6 | TI Palliat* OR AB Palliat* |
| S7 | TI ( ((terminal* or "end stage*" or endstage* or "advanced stage*" or "late stage*") N2 (disease* or ill* or care* or caring or treatment* or period* or nurs* or patient*)) ) OR AB ( ((terminal* or "end stage*" or endstage* or "advanced stage*" or "late stage*") N2 (disease* or ill* or care* or caring or treatment* or period* or nurs* or patient*)) ) |
| S8 | TI ( ((incurabl* or irreversibl*) N0 ill*) ) OR AB ( ((incurabl* or irreversibl*) N0 ill*) ) |
| S9 | TI ( eol or "end of life" or dying ) OR AB ( eol or "end of life" or dying ) |
| S10 | TI ( ((advance*) N2 (plan* or directive*)) ) OR AB ( ((advance*) N2 (plan* or directive*)) ) |
| S11 | TI hospice* OR AB hospice* |
| S12 | TI ( (("life limit*" or "life threatening") N2 (disease* or condition* or illness*)) ) OR AB ( (("life limit*" or "life threatening") N2 (disease* or condition* or illness*)) ) |
| S13 | TI ( ((advance* N0 (disease* or illness*)) ) OR AB ( ((advance* N0 (disease* or illness*)) ) |
| S14 | (MH "Neoplasms") |
| S15 | (MH "Cerebral Palsy") |
| S16 | (MH "Brain Injuries") |
| S17 | (MH "Spinal Cord Injuries") |
| S18 | TI ( cancer* or neoplasm* or "cerebral pals*" or "brain injur*" or "spinal cord injur*" ) OR AB ( cancer* or neoplasm* or "cerebral pals*" or "brain injur*" or "spinal cord injur*" ) |
| S19 | S1 OR S2 OR S3 OR S4 OR S5 OR S6 OR S7 OR S8 OR S9 OR S10 OR S11 OR S12 OR S13 OR S14 OR S15 OR S16 OR S17 OR S18 |
| S20 | (MH "Telemedicine") OR (MH "Telerehabilitation") OR (MH "Telepsychiatry") OR (MH "Telehealth") OR (MH "Teledentistry") OR (MH "Telepathology") OR (MH "Teleradiology") OR (MH "Telenursing") OR (MH "Telenutrition") |
| S21 | (MH "Remote Consultation") |
| S22 | (MH "Internet-Based Intervention") OR (MH "Digital Technology") OR (MH "Digital Health") |
| S23 | (MH "Videoconferencing") OR (MH "Webcasts") |
| S24 | (MH "Telecommunications") OR (MH "Wireless Communications") OR (MH "Teleconferencing") |
| S25 | (MH "Telephone") OR (MH "Cellular Phone") OR (MH "Text Messaging") OR (MH "Smartphone") |
| S26 | (MH "Computers, Portable") OR (MH "Computers, Hand-Held") OR (MH "Microcomputers") OR (MH "Macintosh Microcomputers") OR (MH "IBM Compatible Microcomputers") OR (MH "User-Computer Interface") OR (MH "Computer Assisted Instruction") |
| S27 | TI ( ((wearable or wireless or portable) N1 (technolog* or electronic* or device*)) or (digital N1 medicine) or ((technolog*) N1 (remote or health)) or (remote N1 care) or ((mobile or internet or electronic* or robot* or remote or virtual or wireless) N1 (consultation* or app* or device* or rehab* or communicat* or team*)) ) OR AB ( ((wearable or wireless or portable) N1 (technolog* or electronic* or device*)) or (digital N1 medicine) or ((technolog*) N1 (remote or health)) or (remote N1 care) or ((mobile or internet or electronic* or robot* or remote or virtual or wireless) N1 (consultation* or app* or device* or rehab* or communicat* or team*)) ) |
| S28 | TI ( telecommunicat* or "tele communicat*" or teleconferenc* or "tele conferenc*" or app or apps or "mobile based" or "Short Message* Service*" or sms or textmessag* or "text messag*" or texting or videoconferenc* or "video conferenc*" or webconferenc* or "web conferenc*" or webcast* or "web cast*" or webinar* or "web based" or "web camera*" or "web application*" ) OR AB ( telecommunicat* or "tele communicat*" or teleconferenc* or "tele conferenc*" or app or apps or "mobile based" or "Short Message* Service*" or sms or textmessag* or "text messag*" or texting or videoconferenc* or "video conferenc*" or webconferenc* or "web conferenc*" or webcast* or "web cast*" or webinar* or "web based" or "web camera*" or "web application*" ) |
| S29 | TI ( smartphone* or "smart phone*" or cellphone* or "cell phone*" or mobilephone* or "mobile phone*" or "personal digital assistant*" or palmpilot* or "palm pilot*" or smarthome* or "smart home*" or touchscreen* or "touch screen*" or "high tech*" or hightech* ) OR AB ( smartphone* or "smart phone*" or cellphone* or "cell phone*" or mobilephone* or "mobile phone*" or "personal digital assistant*" or palmpilot* or "palm pilot*" or smarthome* or "smart home*" or touchscreen* or "touch screen*" or "high tech*" or hightech* ) |
| S30 | TI ( telemedicin* or "tele medicin*" or telehealth* or "tele health*" or telecare* or "tele care*" or telecari* or "tele cari*" or emedic* or "e medic*" or ehealth* or "e health*" or mhealth* or "m health*" or ehomecare* or "e homecare*" or "e home care*" or telehomecare or "tele homecare" or "tele home" or telenurs* or "tele nurs*" or teletherap* or "tele therap*" or telerehab* or "tele rehab*" or erehab* or "e rehab*" or teleconsultation* or "tele consultation*" or videoconsultation* or "video consultation*" or telemonitor* or "tele monitor*" or "connected car*" ) OR AB ( telemedicin* or "tele medicin*" or telehealth* or "tele health*" or telecare* or "tele care*" or telecari* or "tele cari*" or emedic* or "e medic*" or ehealth* or "e health*" or mhealth* or "m health*" or ehomecare* or "e homecare*" or "e home care*" or telehomecare or "tele homecare" or "tele home" or telenurs* or "tele nurs*" or teletherap* or "tele therap*" or telerehab* or "tele rehab*" or erehab* or "e rehab*" or teleconsultation* or "tele consultation*" or videoconsultation* or "video consultation*" or telemonitor* or "tele monitor*" or "connected car*" ) |
| S31 | TI ( "internet based intervention*" or ((digital or online) N1 intervention*) ) OR AB ( "internet based intervention*" or ((digital or online) N1 intervention*) ) |
| S32 | S20 OR S21 OR S22 OR S23 OR S24 OR S25 OR S26 OR S27 OR S28 OR S29 OR S30 OR S31 |
| S33 | (MH "Infant") OR (MH "Adolescence") OR (MH "Child, Preschool") OR (MH "Child") OR (MH "Infant, Newborn") |
| S34 | TI ( (pediatric* or paediatric* or peadiatric* or neonatal* or "neo natal"* or neonate* or newborn* or "new born*" or infant* or baby or babies or toddler* or child* or kid or kids or girl or girls or boy or boys or minors or underage* or "under age*" or teen* or youth* or youngster* or adolescent* or adolescence or preadoles* or "pre adoles*" or juvenil* or puber* or pubescen* or "pre puber*" or prepuber* or prepubescen* or "pre pubescen*" or schoolchild* or preschool* or (young N1 (adult* or man or men or woman or women or person* or people))) ) OR AB ( (pediatric* or paediatric* or peadiatric* or neonatal* or "neo natal"* or neonate* or newborn* or "new born*" or infant* or baby or babies or toddler* or child* or kid or kids or girl or girls or boy or boys or minors or underage* or "under age*" or teen* or youth* or youngster* or adolescent* or adolescence or preadoles* or "pre adoles*" or juvenil* or puber* or pubescen* or "pre puber*" or prepuber* or prepubescen* or "pre pubescen*" or schoolchild* or preschool* or (young N1 (adult* or man or men or woman or women or person* or people)))) |
| S35 | S33 OR S34 |
| S36 | S19 AND S32 AND S35 |
| S37 | S19 AND S32 AND S35 |
| S38 | S19 AND S32 AND S35  Limiters - Publication Date: 20180101-20231231 |

Database: Web of Science Core Collection (1987-present)

| 1 | TS=(Palliat*) |
| --- | --- |
| 2 | TS=((terminal* or "end stage*" or endstage* or "advanced stage*" or "late stage*") NEAR/2 (disease* or ill* or care* or caring or treatment* or period* or nurs* or patient*)) |
| 3 | TS=(((incurabl* or irreversibl*) NEAR/0 ill*)) |
| 4 | TS=((eol or "end of life" or dying)) |
| 5 | TS=(((advance*) NEAR/2 (plan* or directive*))) |
| 6 | TS=(Hospice*) |
| 7 | TS=((("life limit*" or "life threatening") NEAR/2 (disease* or condition* or illness*))) |
| 8 | TS=((advance* NEAR/0 (disease* or illness*))) |
| 9 | TS=((cancer* or neoplasm* or "cerebral pals*" or "brain injur*" or "spinal cord injur*")) |
| 10 | #9 OR #8 OR #7 OR #6 OR #5 OR #4 OR #3 OR #2 OR #1 |
| 11 | TS=((((wearable or wireless or portable) NEAR/1 (technolog* or electronic* or device*)) or (digital NEAR/1 medicine) or ((technolog*) NEAR/1 (remote or health)) or (remote NEAR/1 care) or ((mobile or internet or electronic* or robot* or remote or virtual or wireless) NEAR/1 (consultation* or app* or device* or rehab* or communicat* or team*)))) |
| 12 | TS=((telecommunicat* or "tele communicat*" or teleconferenc* or "tele conferenc*" or app or apps or "mobile based" or "Short Message* Service*" or sms or textmessag* or "text messag*" or texting or videoconferenc* or "video conferenc*" or webconferenc* or "web conferenc*" or webcast* or "web cast*" or webinar* or "web based" or "web camera*" or "web application*")) |
| 13 | TS=((((electronic or mobile or digital) NEAR/0 (health)) or ((information or communicat*) NEAR/0 (technolog*)) or "application of technolog*")) |
| 14 | TS=((smartphone* or "smart phone*" or cellphone* or "cell phone*" or mobilephone* or "mobile phone*" or "personal digital assistant*" or palmpilot* or "palm pilot*" or smarthome* or "smart home*" or touchscreen* or "touch screen*" or "high tech*" or hightech*)) |
| 15 | TS=((telemedicin* or "tele medicin*" or telehealth* or "tele health*" or telecare* or "tele care*" or telecari* or "tele cari*" or emedic* or "e medic*" or ehealth* or "e health*" or mhealth* or "m health*" or ehomecare* or "e homecare*" or "e home care*" or telehomecare or "tele homecare" or "tele home" or telenurs* or "tele nurs*" or teletherap* or "tele therap*" or telerehab* or "tele rehab*" or erehab* or "e rehab*" or teleconsultation* or "tele consultation*" or videoconsultation* or "video consultation*" or telemonitor* or "tele monitor*" or "connected car*")) |
| 16 | TS=(("internet based intervention*" or ((digital or online) NEAR/1 intervention*))) |
| 17 | #11 OR #12 OR #13 OR #14 OR #15 OR #16 |
| 18 | TS=((pediatric* or paediatric* or peadiatric* or neonatal* or "neo natal"* or neonate* or newborn* or "new born*" or infant* or baby or babies or toddler* or child* or kid or kids or girl or girls or boy or boys or minors or underage* or "under age*" or teen* or youth* or youngster* or adolescent* or adolescence or preadoles* or "pre adoles*" or juvenil* or puber* or pubescen* or "pre puber*" or prepuber* or prepubescen* or "pre pubescen*" or schoolchild* or preschool* or (young NEAR/1 (adult* or man or men or woman or women or person* or people)))) |
| 19 | #10 AND #17 AND #18 |
| 20 | #10 AND #17 AND #18 and 2018 or 2019 or 2020 or 2021 or 2022 or 2023 (Publication Years) |

Database: ASSIA

| S1 | noft(palliat* OR eol OR "end of life" OR dying OR hospice* OR cancer* OR neoplasm* OR "cerebral pals*" OR "brain injur*" OR "spinal cord injur*") OR noft(((terminal* OR "end stage*" OR endstage* OR "advanced stage*" OR "late stage*") NEAR/3 (disease* OR ill* OR care* OR caring OR treatment* OR period* OR nurs* OR patient*))) OR noft(((incurabl* OR irreversibl*) NEAR/0 ill*)) OR noft((advance* NEAR/3 (plan* OR planning OR directive*))) |
| --- | --- |
| S2 | noft((((wearable OR wireless OR portable) NEAR/2 (technolog* OR electronic* OR device*)) OR (digital NEAR/2 medicine) OR (technolog* NEAR/2 (remote or health)) OR (remote NEAR/2 care) OR ((mobile OR internet OR electronic* OR robot* OR remote OR virtual OR wireless) NEAR/2 (consultation* OR app* OR device* OR rehab* OR communicat* OR team*)))) OR noft((telecommunicat* OR "tele communicat*" OR teleconferenc* OR "tele conferenc*" OR app OR apps OR "mobile based" OR "short message* service*" OR sms OR textmessag* OR "text messag*" OR texting OR videoconferenc* OR "video conferenc*" OR webconferenc* OR "web conferenc*" OR webcast* OR "web cast*" OR webinar* OR "web based" OR "web camera*" OR "web application*")) OR noft((((electronic OR mobile OR digital) NEAR/0 health) OR ((information OR communicat*) NEAR/0 technolog*) OR "application of technolog*")) OR noft((smartphone* OR "smart phone*" OR cellphone* OR "cell phone*" OR mobilephone* OR "mobile phone*" OR "personal digital assistant*" OR palmpilot* OR "palm pilot*" OR smarthome* OR "smart home*" OR touchscreen* OR "touch screen*" OR "high tech*" OR hightech*)) OR noft((telemedicin* OR "tele medicin*" OR telehealth* OR "tele health*" OR telecare* OR "tele care*" OR telecari* OR "tele cari*" OR emedic* OR "e medic*" OR "ehealth*" OR "e health*" OR mhealth* OR "m health*" OR ehomecare* OR "e homecare*" OR "e home care*" OR telehomecare OR "tele homecare" OR "tele home" OR telenurs* OR "tele nurs*" OR teletherap* OR "tele therap*" OR telerehab* OR "tele rehab*" OR erehab* OR "e rehab*" OR teleconsultation* OR "tele consultation*" OR videoconsultation* OR "video consultation*" OR telemonitor* OR "tele monitor*" OR "connected car*")) OR noft((("internet based intervention*" OR (digital OR online)) NEAR/2 intervention*)) |
| S3 | noft((pediatric* OR paediatric* OR peadiatric* OR neonatal* OR "neo natal*" OR neonate* OR newborn* OR "new born*" OR infant* OR baby OR babies OR toddler* OR child* OR kid OR kids OR girl OR girls OR boy OR boys OR minors OR underage* OR "under age*" OR teen* OR youth* OR youngster* OR adolescent* OR adolescence OR preadoles* OR "pre adoles*" OR juvenil* OR puber* OR pubescen* OR "pre puber*" OR prepuber* OR prepubescen* OR "pre pubescen*" OR schoolchild* OR preschool* OR (young NEAR/2 (adult* OR man OR men OR woman OR women OR person* OR people)))) |
| S4 | [S1] AND [S2] AND [S3] |
| S5 | [S1] AND [S2] AND [S3] Limit 2018-2023 |
