# Supplementary material for: Health Technologies and Infrastructures for Supporting Home-Based Pediatric Palliative Care: Scoping Review
Source: J Med Internet Res. 2025 Dec 8;27:e70687. doi: 10.2196/70687 (PMC12723365; doi:10.2196/70687)
Supplement: Multimedia Appendix 4 [file jmir_v27i1e70687_app4.docx]

**Multimedia Appendix 4** Description of health technologies and infrastructures

| **Author,**  **year** | **Aim of health technology** | **Development of health technology** | **Users of health technology** | **Health technology, follow-up service, mode of delivery** | **Infrastructure** |
| --- | --- | --- | --- | --- | --- |
| Archer et al., 2021 [73] | To improve patient/carer/clinician communication, to allow families to identify, describe, prioritize and monitor the issues that most impact on their quality of life, and to share this information with their health and social care team | Not reported (NR) | Families,  children (0-18 years), healthcare professionals (HCPs) (hospice nurse, pediatric palliative care physician pediatric palliative care physician, pediatric palliative service) | - Video to provide specialist consultations directly to patients and their caregivers at home; symptom management, emotional support. Synchronous delivery - In-person visit hospice nurse within 48 h after hospital discharge. Inpatient pediatric palliative care physician joint visit using video (FaceTime). Synchronous delivery - Website (MyQuality) allows families to identify, describe, prioritize, monitor and share issues that most impact on their quality of life. Asynchronous delivery | - Internet - Logitech video - Facetime - Zoom - Bespoke website (free of charge) - Access to data controlled by patient/carer |
| Bird et al., 2022 [48] | To facilitate home-based care management decision-making | Designed with hospital-based HCPs, families, and home-based HCPs | Families, HCPs (hospital-based, home-based) | - DigiComp Kids use hospital HCP portal and home-based kit to communicate - Hospital-based HCPs team review biometric data and health information submitted by families and home-based HCPs and send health information to home-based kits to facilitate home-based care management decision-making - Hospital-based HCPs can review submitted patient vital sign measurements, photos, and survey responses; configure individual vital sign parameters and alerts for each patient; send and receive secure messages with family members and home-based HCPs; send health-related documents to families and home-based HCPs; video calls with families and home-based HCPs; document patient care information directly within the hospital HCP portal - Asynchronous and synchronous delivery | - Cloud DX Connected Health System - Hospital HCP portal - Home-based kit: Samsung tablet, Bluetooth-enabled pulse oximeter with heart rate monitoring capabilities, dual tympanic-temporal infrared thermometer |
| Bradford et al., 2021 [34] | To reduce the total symptom burden experienced by children receiving treatment for childhood cancer | Iteratively developed with HCPs, children and caregivers (oncology, medical, nursing, allied health). Clinical reference group to choose e-proms, develop algorithms to determine threshold for symptoms measured, cognitive interviews with children and caregivers to ensure content validity, evidence-based symptom self-management recommendations were drafted, wording reviewed by 2 adolescents | Children aged 4-18 years; HCPs | - RESPONSE mobile phone app: weekly monitoring of symptoms using a validated, child friendly electronic patient reported outcome measure (ePROM) (SSPedi). Stratified alerts for symptoms that reach pre-determined thresholds requiring intervention (green: no concern; amber: discussion with HCPs next clinical visit; red: immediate response, parents phone hospital) - Evidence-based symptom management recommendations - Graphical displays of SSPedi information enabling symptom trends to be visualized over time - HCPs manage users, facilitate push notifications (reminders to complete ePROMs), tracking in a convenient format) - Asynchronous delivery | - Mobile phone app (access children, parents) - App database stored on a secure and private cloud server within Australia - Web-based portal for HCPs - Data are not linked to medical records |
| Brock et al., 2018 [35] | To facilitate more timely care based on the patient reported outcomes (PRO) reports from the children | NR | Child, caregivers, HCP | - Online or computer-based tools and mobile apps to collect PRO from the child or caregivers - HCP can receive alerts and act accordingly - Various services with various follow-up possibilities - Asynchronous and synchronous delivery | Various needs, based on the relevant technology, such as smart phones, tablets, computers, internet or WiFi access |
| Canter, 2019 [59] | To deliver psychosocial care in a flexible manner that accommodates the needs of  the family during cancer treatment | NR | Caregivers, HCP (life with cancer therapists) | - The Electronic Surviving Cancer   Competently Intervention Program (eSCCIP): hybrid intervention  (self‐guided, web‐based content and telehealth) for caregivers   - Life with Cancer therapists deliver eSCCIP - Mode of delivery NR | NR |
| Canter et al., 2019 [60] | To support psychosocial needs in parents of children with cancer | Participatory process: creating video discussion and content; developing the initial prototype; Think Aloud testing; beta testing. Close collaboration parents, review by professionals | Parents of children with cancer, HCP (study interventionist) | - eSCCIP: hybrid eHealth intervention or guided behavioral intervention technology. Contains four self-directed sessions, called modules, including an introductory moduls. through the set modules - Asynchronous and synchronous delivery | - Designed to be utilized anywhere with internet access or mobile data is available (e.g. laptops, mobile phones) |
| Canter et al., 2022 [61] | To deliver psychosocial interventions | Developed using best practices for user-centered design work, and  preliminary results from Think Aloud and beta testing. Parents provided feedback at all steps of intervention design and initial testing | Parents individually or as dyad, HCPs (licensed psychologist or social worker) | - eSCCIP: brief, hybrid intervention for parents that includes 4 online modules (an orientation module and 3 core content modules) - Modules are interactive, featuring a mix of video content, engaging skills practice (game like activities, interactive examples, and free-response questions) - Telehealth sessions with an interventionist (e.g., licensed psychologist or social worker) - Asynchronous and synchronous delivery | - Interventionist - Video content - Web program (eSCCIP) - Intervention manual - Hotspot to reduce Wi-Fi barriers |
| Canter et al., 2023 [62] | To reduce negative psychosocial sequelae following pediatric cancer and improve family functioning | Developed using best practice methodology from the digital health literature, stakeholders, and small-scale beta testing. The intervention was refined using think aloud usability testing, collaboration with community. The theory and content of eSCCIP were adapted from 2 prior in-person interventions for parents and caregivers of children with cancer: the Surviving Cancer Competently Intervention Program and the Surviving Cancer Competently Intervention Program—Newly Diagnosed | Parents/caregivers of child with cancer (0-18 years), HCP (telehealth (TH) guide) | - eSCCIP: web-based, self-directed cores designed for caregivers/parents to complete over 1 month during their child’s active cancer treatment or shortly after completion of treatment - The self-guided web-based cores are a mix of didactic video content, multifamily video discussion groups featuring parents of children with cancer, and hands-on web-based activities. Each core is complemented by a brief TH follow-up with a TH guide - All web-based activities are securely captured by the program, allowing the guide to view data entered by participants (e.g., free text responses) and integrate participant responses into TH follow-up sessions - Asynchronous and synchronous delivery | - Video content - Web program - Intervention materials available in English and Spanish language - Intervention is maintained in the Research Infrastructure - Accesses through a secure portal - Access to internet (cellular data or borrowed institutional device) |
| Castro et al., 2021 [69] | To reduce morbidity/mortality and to improve outcomes | NR | Caregivers of children with  complex congenital heart diseases, HCPs (type NR) | - A virtual-face mixed model program, cellphone with a specific app, oximeter, scale, audio and video calls. Caregivers received training and education - Follow-up using audio/video calls, regular cardiology checkups. Monitoring data reviewed in a control center. Continuous education reinforcements done at every contact with caregivers and through social networks/website - Asynchronous and synchronous delivery | - Cellphone - App - Oximeter - Scale - Control center |
| Dreher et al., 2023 [36] | To reduce treatment burden | NR | Children with leukemia, families, HCPs | - ePROtect mobile application for continuous health monitoring - Video consultations for routine check-ups during maintenance therapy - Synchronous delivery | - ePROtect symptom reporting platform |
| Ellis & Lindley, 2020 [49] | To deliver respite and end of life care | Senior leadership team managed the delivery of the virtual hospice. Review and development of several policies and procedures. Key outcome measures of telehospice were developed. Personal and team restructuring took place | Children, families  HCPs, social worker, volunteers | - Videoconference platform, virtual outpatient clinic including waiting rooms, secure patient encounter rooms - Telephone: Kindness calls from HCPs inquire about the families. Opportunity to set up a virtual appointment with nursing, pharmacy, or physicians. - Volunteer-led services such as friendship calls, live storytelling, live craft, art and baking sessions, and letter writing to children and young people - Synchronous delivery | - Internet - Videoconference platform Near me/Attend anywhere - Secure platform |
| Foster et al., 2021 [71] | To enrich routine home surveillance during the interstage period | Focus groups were conducted with the interstage program  providers and staff to develop a program logic model and  care model diagram. A care model diagram was  then created to further describe the integration of telemedicine  with in-person care | Parents of infants with single ventricle physiology,  HCPs (nurses, advanced practice nurses) | - Home monitoring equipment: scale, pulse oximeter, binder, iPad to conduct weekly video visits with nurse or advanced practice nurse coordinator on teleIHM platform - Daily teleIHM data collection on e.g. heart rate, oxygen saturations, weight entered by parents - Parents can share video/photo with HCPs - Supplementary apps; to access their infant’s electronic health record; for meditation and stress relief; educational material (congenital heart conditions, cardiac catheterization) - Monthly clinic visits - Asynchronous and synchronous delivery | - Tablet - Secure videoconference app (Polycom® RealPresence® system, Santa Cruz, CA) - teleIHM platform (LocusHealth ®, Charlottesville, VA) - App access infant’s electronic health record (MyChart © Epic Systems Corporation, Verona, WI) - App for meditation and stress relief (Headspace © Headspace, Inc; Calm ©, Calm.com, Inc.) - App providing educational material on congenital heart conditions (Heartpedia ©, Cincinnati Children’s Hospital Medical Center) - Informational pop-up on cardiac catheterization by the care team - Pre-written materials (e.g., instructions, references) in English and Spanish |
| Grootenhuis, 2020 [63] | To provide children and parents with psychosocial interventions to support them in coping with the consequences of childhood  cancer and improve their quality of life and lessen the psychosocial  consequences. | Use evidence-based  techniques from cognitive behavioral therapy | Adolescents, family members, HCPs (psychologist, basic psychologist), social worker | - Online sessions consist of 6-8 90-minute chat group sessions in a chat box, homework between sessions - Booster session 6 months after the start of intervention - 3-6 participants per group, 2 course leaders, additional information parents in online environment - Synchronous delivery | - Secured chat box |
| Holmen et al., 2020 [26] | Easier access to services through video and increased quality of life through PRO | NR | Children (age NR), caregivers of children aged 0-18 years, HCPs (medical, nursing, and allied  Health) and social worker | 4 different solutions identified   - Home Telehealth Program using videoconferences (Australia). Synchronous delivery - MyQuality online with PRO for mutual sharing in a website (the UK). Asynchronous delivery - Videoconference through computer (Australia). Synchronous delivery - iPad for videoconferences for clinical review, case conferences, bereavement follow-up; email, internet search, socialization apps, relaxation and mood   apps, and children’s movies and electronic books. Asynchronous and synchronous delivery | - iPad - Laptop computer with external webcams and headset - Individualized apps - Internet - MyQuality online on a website |
| Hunter et al., 2018 [37] | To improve pain management | NR | Children (8-18 years), HCPs | - A web-based application, Pain Buddy, a validated pain and symptom diary, cognitive and behavioral coping skills training, an electronic tool for communicating with HCPs, and a three-dimensional avatar to guide the child through the program - 8 weeks with symptom diary/pain-buddy twice daily - Asynchronous delivery | - Web-based app |
| Jibb et al., 2018 [39] | To enhance pain management | Development followed approach by the UK Medical Research Council; pain management evidence base for  the intervention established using systematic review and expert consensus  conference; software prototype developed; adapted to the needs of adolescents with cancer using iterative cycles of usability testing; multicenter pre-post  test pilot study | Adolescents with cancer (12-18 years HCPs (pediatric oncology-trained  registered nurse) | - The Pain Squad+ smartphone app based on gamification, where the adolescents played to be law-enforcement officer - Scoring of pain twice daily. If pain reported on any assessment adolescents received real-time pain self- management advice from the app according to an evidence-based investigator-developed algorithm, another pain assessment have to be completed within 1 h - Scoring above a threshold yielded alert to a nurse who contacted adolescent and medical team to discuss and initiate intervention - Asynchronous and synchronous delivery | - Apple iPhone 6 with the Pain Squad+ smartphone app |
| Jibb et al., 2020 [38] | To provide adolescents with cancer real-time pain self-management support | App was developed using a phased-centered and user-centered approach, been testes in 1-group, baseline-poststudy pilot. Adolescents with cancer were involved in all stages of the development and evaluation of app | Adolescents with cancer (12-18 years) receiving cancer directed therapy, HCP (pediatric oncology-trained registered nurse) | - The Pain Squad+ smartphone app, real-time pain treatment support. The app is ‘gamified’; users play the role of superheroes who receive rewards for adherence to pain assessment and treatment - When pain is reported self-management advice is presented according to standardized clinical care algorithm. Advice is based on a library of pharmacological, psychological and physical advice - 3 consecutive moderate-to-severe reports of pain intensity trigger email to a nurse who contacts the adolescent and/or their health care team to discuss and initiate HCP driven intervention - Asynchronous and synchronous delivery | - Smartphone app available from Apple app store and Google play store - Smartphone (iPhone/   Android phone   - Can loan smartphone - Library of advice - Encrypted and wireless transfer to secure server (SickKids) - Platform for HCPs |
| Lai et al., 2023 [40] | To increase awareness of symptoms, and to accurate measure and record symptoms | Symptom Monitoring and Systematic Assessment and Reporting System in Young Survivors (SyMon-SAYS) was modified from a self-monitoring model, SyMon-SAYS pilot study and literature. User involvement NR | Children (aged 8 to 17 years) living with cancer, parents, guardian, HCPs, | - SyMon-SAYS, an EHR-based system, a self-monitoring of symptoms via weekly systematic symptom assessments and graphic reports - Self-management by directing patients or parents to web-based resources for strategies to manage symptoms - Symptom scores automatically monitored and reported to HCPs. When symptom score exceeds the preset severity threshold an email alert is sent to HCPs who determine whether a call is needed - Asynchronous and synchronous delivery | - SyMon-SAYS - Epic MyChart patient portal - Device with internet (smartphone, tablet, or computer) - Children’s medical records - web-based resources developed by clinicians for appropriate strategies |
| LeBlanc et al. 2024 [58] | To augment home monitoring of high-risk infants including single ventricle patients in the “interstage” period | Developed with parent input via a series of focus groups. First tested by a small number of collaborators | Parents, HCPs | - Educational materials in video and PDF modalities, customizable center information, diagnostic summary, chat feature accessible to both parents and providers - Parents encouraged to input daily growth and feeding data, vital signs, medication compliance. They can track progress using the app’s graphical features and receive rewards and reminders via the app to encourage appropriate data submission - A red flag monitoring survey and a neurodevelopmental survey. A red flag event (any score above 0) triggers alert, the care team then contact parents directly to evaluate symptoms and to triage needs - Asynchronous delivery and synchronous delivery | - KidsHeart app - The Pattern HealthTM mobile app platform (Pattern Health Technologies, Inc., Durham, NC) - Access to the app via the care team - Provide digital consent upon downloading the app |
| Liu et al., 2022 [41] | To assist in symptom management | Based on parent and expert interviews, children's symptoms investigation, a multidisciplinary research team worked collaboratively in developing the main module and prototype. Several discussions and improvements of the prototype | Children with cancer (5–10 years), parents, HCPs (nurses, doctors) | - WeChat Mini Program “Child SMILE”: symptom assessment and management module, information and emotional support module, communication module among the parents and HCPs and personal information module - The communication module: One platform for parents to communicate with each other; HCPs also have the opportunity to join different topics, questions related to child's adverse symptoms, there were HCPs to answer questions or give the advice to the hospital - Asynchronous delivery | - Smartphone - WeChat App - Web-based administration portal - Cloud storage server |
| Marc-Aurele et al., 2020 [51] | To provide palliative care to children with complex chronic conditions who face many barriers in their ability to travel to clinic | NR | Children with complex chronic conditions (10 months to 19 years old), family, HCPs (palliative nurse, physician) | - Telemedicine visit using Zoom - Homebased palliative nurse, with an ongoing patient and family relationship, spends 30 minutes in patient’s home before each telemedicine visit to assess patient and strategize with family what to discuss with physician. Nurse shares vital signs and detailed physical exam to supplement physician’s visual assessment - Synchronous delivery | - Laptop - Encrypted institutional pro-Zoom account |
| McLeod & Star, 2021 [72] | To maximise therapeutic outcomes for the child and their parent | NR | Children with complex congenital heart disease, spinal muscular atrophy or Lafora progressive myoclonus epilepsy (4, 5 or 17 years), parents, music therapist | - ICT platform accessed via web or app, therapists use laptop with Wi-Fi internet connection. Pexip was emailed parents - A ‘test run’ was offered prior to the first session. A basic overview of what the session could entail was provided to families. Conversations to identify goals and priorities which informed session planning. a supportive adult needed to be present, child participation was encouraged, session duration tailored for the child’s developmental age - Music therapist sang and played acoustic guitar for most songs, facilitated playing of recorded music - Synchronous delivery | - ICT platform (Pexip®) a secure, cloud-native platform - Web or app to access ICT platform with high quality audio and visual capabilities - Laptop - Wi-Fi internet connection |
| Mehdizadeh et al., 2022 [64] | To support children with cancer and their caregivers | Adopting a user-centered design approach a mobile app was developed: requirement analysis by conducting review and focus groups for children, parents, HCPs; conformity analysis with end-user’s end design team; preparing educational content using guidelines; app prototyping; preliminary evaluation using think-aloud; app development | Children with  acute lymphocytic leukemia (7-14 years), parents,  HCPs | - Mobile app (CanSelfMan), gamification elements and 5 main modules: knowledge base, self-management tips, self-assessment report (standard Edmonton Symptom Assessment System–Revised questionnaire), ask questions and reminders - Ask questions by completing and sending a form. Response time to the questions was maximum 24 h - The oncologist could view the self-assessment reports, make suggestions, send messages. Web-based dashboard was for oncologists for direct communication - Asynchronous delivery | - Mobile app (CanSelfMan) 2 different versions for children and parents - Web-based dashboard for oncologist |
| Mehdizadeh et al., 2023 [65] | To support children with cancer and their parents/ caregivers, by providing access to up-to-date and reliable information about cancer and information on how to deal with and manage symptoms related to cancer | NR | Children with acute lymphocytic leukemia (at least 7 years), parents, HCPs | - CanSelfMan is a self-management system for children with cancer, a single android app for both children and their parents/ caregivers, web-based dashboard for oncologists - 5 modules; cancer knowledge, self-management recommendations, symptom management, self-assessment questionnaire of symptoms - Oncologists see results of patient assessments, questions, and answers to patients’ questions - Asynchronous delivery | - Android smartphone (version 8 to 11) - Android app - Web-based dashboard - Cloudy platform |
| Meryk et al.,  2025 [57] | To reduce the necessity for in-person visits while enhancing the overall patient experience in terms of quality and satisfaction | NR | Children, family, pediatric oncologist | - Outpatient services provided by local physicians, diagnostic procedures conducted local hospital or by the general practitioner - Video consultation of the same day through the ePROtect app - The findings from the clinical examination reviewed and lead to adjustments of maintenance therapy based on blood results - Appointments booked through the app and the family was responsible for arranging the next appointment with the local physicians - Synchronous delivery | - App - Patients and families were trained in the use of the ePROtect software |
| Meryk et al., 2021 [43] | To help overcome obstacles in the routine measurement of patient-reported symptoms in the treatment of childhood cancer | NR | Child (10-year-old), HCPs | - Daily self-reporting via ePROtect an interface based on the CHES software (computer-based evaluation system) - ePROtect includes 6-8 questions adapted from the Pediatric Quality of Life Inventory (PedsQL) 3.0 Cancer Module - The monitoring questionnaire assesses symptom burden on the previous day - Aberrant symptom scores trigger to HCPs who contact parents by phone, to confirm the child's self-report - Asynchronous delivery | - CHES software - App |
| Meryk et al., 2021 [42] | To monitor patients’ symptom burden during and after treatment | ePROtect was developed based on the computer-based health evaluation system (CHES). User involvement NR | Children with cancer (5-18 years), HCPs | - Web-based patient portal (ePROtect), animation characters, patient stories of experience during treatment, detailed explanation of the need to assess patients’ HRQoL - Complete daily PROMs, provide specific information on the respective disease, its treatment, necessary medical examinations, the ward plan, and the HCPs. When symptom severity of level 3 or 4 was reported, automated alarm for HCPs was triggered and interventions undertaken. Patient contacted by HCPs when combinations of moderate symptom (level 2) resulted in significant symptom burden and had persisted over a period of 48–72 h - HCP interface to review data from all patients and to integrate it into their care - Asynchronous delivery | - Web-based patient portal - Smartphone, tablet, or computer (no installation required) - iPad provided by HCPs if parents or patient did not have - HCP interface |
| Munoz-Bonet et al., 2020 [50] | To avoid hospital confinement | NR | Tracheostomized ventilator dependent children, parents, HCPs (specialized nurses) | - A high-density data remote monitoring system, (Medlinecare 2.1): monitor patients in real time from the PICU; visual and acoustic medical alert for each patient and parameter. Store and process information for subsequent further evaluation; includes a tele-auscultation and videoconference device via an Internet protocol camera with pan-tilt-zoom which can be controlled from the PICU - Patients monitored during rest periods (day and night) and whenever necessary, care was provided - Specialized nurses have telemedicine visit at hospital with school and/or at home including respite care, and varies according to the basic needs and contingencies of each patient - Asynchronous delivery and synchronous delivery | - Monitoring system, Medlinecare 2.1 (Medical Online Technolog SL, Valencia, Spai) including tele auscultation, video-conference device - Pulse oximeter - Internet protocol camera with pan-tilt- zoom - Internet - Hospital control system - SMS alarms - Smartphone (HCPs) |
| Novrianda et al., 2023 [44] | To identify symptoms and provide recommendation strategies to manage the symptom | Development of app through multiple phases: development based on need assessment; creation of content based on literature review; develop prototyping; expert review and feedback on content; usability testing by end-user. Parents, HCPs, IT expert participated | Children, parents, HCPs | - Mobile health app (Chemo Assist for Children): 7 modules: patient demographic data, primary health data, regulation of caloric and fluid requirements, symptom identification with *Symptom Screening in Pediatrics* (SSPedi) tools, chemotherapy-related symptoms, information, and consultation - Asynchronous delivery | - Mobile health app (Chemo Assist for Children) - designed to be used when an internet connection is unavailable, or the network is in inadequate condition |
| Phipps et al., 2020 [66] | To help parents manage the demands of caring for a child with cancer | Development followed a user-centered design process: focus groups to obtain parent perspectives; initial website prototype was reviewed by representative users. Feedback from this review was used by the developers, and program changes were updated in a stepwise incremental process | Parents of children with cancer, counselor | - Web-based, bright ideas via mobile device: modeling videos, interactive activities and homework/work-sheet tool. Videos presenting steps of problem-solving via the interaction between clinician and parent, 4 fotnovellas depicting parents working through typical problems encountered. A counselor interface for web-users - Participants access at least weekly to view videos and fotonovellas, work through ≥ 1 personal problem(s) - Counselors track progress through lessons and viewed completion status/ follow-up as necessary - Asynchronous delivery | - Lap/desktop, tablet or cellphone - Counselor interface for web-users - Content videos and fotonovellas |
| Schultz et al., 2021 [70] | To enhance transparency and improve clinical outcomes | Based on EPIC. User involvement NR | Parents of children with cancer, HCPs | - MyNemours (EPIC-based): Caregivers access to portions of child’s medical records: problem lists, medication list, laboratory and radiology test results, ability to schedule appointments, refill prescriptions, ask the care team questions, print immunization records - Asynchronous delivery | - Patient portal, MyNemours within Epic’s MyChart platform - Online link - Portal account - Internet required |
| Sengul & Toruner, 2020 [67] | To increase coping and quality of life, and decrease stress | NR | Children with cancer (9-18 years), families,  coaches | - Technology‑based psychosocial motivational program for 10 weeks: web‑based training, mobile video coaching interviews, counseling, mobile messages, children’s story reading and writing - A mobile face‑to‑face coaching interview with children conducted once a week for 45–60 min (total of nine coaching interviews), offer parents 2 mobile online video interviews and 24/7 telephone counseling services for both children and families - Synchronous and asynchronous delivery | - Internet access on smartphone or computer - WhatsApp - Website - Video contents which appeal to children   of different ages and parents |
| Senguttuvan et al., 2022 [68] | To manage patients | NR | Families, HCPs (doctors), | - Teleconsultation with doctor, frequency NR - Synchronous delivery | - NR |
| Simon et al., 2021 [46] | To improve pain management and decrease pain in children with cancer | Developed according to secure and controlled processes by an external web design company. Educational information was composed by medical psychologist,  pediatric oncologist specialized in palliative care, and a representative of the center’s Pain and was based on guidelines | Children (8-18), parents (children 0-7 years)  HCPs (hospital’s Pediatric Pain Service) | - KLIK pain monitor app: family version children (aged 8-18) or parents (children aged 0-7) report pain intensity using an 11‐point NRS. Score below set threshold families redirected to interventions on app - HCPs version featured a calling list, with reported pain intensity score, remaining time for contacting the family (based on the set time frame), patient identification number, date of birth, phone number provided by family - HCPs contact families if pain score is above set threshold - Asynchronous and synchronous delivery | - KLIK pain monitor app for Apple and Android - 2-factor authentication - Data Protection Impact Assessment - 3 versions of app: parent version (children aged 0–7 years), child version (children aged 8–18 years for which language was adapted and approved by the Dutch children’s cancer association), HCPs version |
| Simon et al., 2024 [47] | To improve pain management and ultimately reduce clinically significant pain of children with cancer at home | The content of  the app was composed by medical psychologists, pediatric oncologists specialized in palliative care, and a representative of the center’s Pain Service and was based on guidelines | Children, families, HCPs | - KLIK pain monitor app: family version families report pain intensity using an 11‐point NRS. The HCP version featured a calling list, with the reported pain intensity score, remaining time for contacting the family (based on the set time frame), a patient identification number, date of birth, a phone number provided by family. Pain scores 1–3, families redirected to educational information page of app - Scores ≥ 4 families called by HCP within set timeframe (within 2 h for scores 4–6 and within 30 min for scores 7–10) - Asynchronous and synchronous delivery | - 2 versions of the app: family version, HCP version - 2‐factor authentication - Access to a smartphone/tablet on which the application could be downloaded - Users of Huawei phones were excluded because US legislation prohibited this company from using Google Mobile Services in Android at the time of study |
| Simon et al., 2021 [45] | To improve pain management of children with cancer in the home setting | NR | Children,HCPs | - KLIK pain monitor app for pain assessment in home-based children’s - cancer care. App provide educational information about pain (management) - Real-time feedback on reported pain scores (0-10) from HCPs - Asynchronous and synchronous delivery | - Mobile app |
| Stagg et al., 2023 [52] | To replace in-person visits and remain care quality | NR | Family caregivers, HCPs | - Epic care companion app in MyChart: families monitored data at home, and uploaded (NR what data). Daily home monitoring. Biweekly, at least one physical consultation replaced with video - Epic software for HCPs to conduct video visits. HCPs reviewed data at met the families biweekly - Asynchronous and synchronous delivery | - Internet access - Device (e.g., smartphone, iPad) - Provided an iPad for those who did not have access to such - Patient portal app - Clinicians used software embedded in the EMR (Epic Systems Corporation; Verona, Wis.) |
| Warniment et al., 2023 [53] | To support transition from hospital to home | Developed by theory and lived transition experiences of families with children with medical complexity. Multi- disciplinary research team developed the intervention | Children, family, HCPs (inpatient team, primary care  outpatient team, home nursing providers) | - The GET2HOME bundle intervention: a telehealth huddle to plan discharge, a visual discharge tracker and a post discharge telehealth huddle - 7 days after discharge a huddle Teams meeting will be conducted following a checklist - Asynchronous and synchronous delivery | - Microsoft Teams to facilitate huddle meetings - My chart bedside (Epic) for data monitoring of the task tracker |
| Weaver et al. 2020 [54] | To support palliative care at home and to inform palliative communication | NR | Family, HCPs (palliative physician, hospice nurse) | - Telehealth solution, not specified: hospice nurse in child’s home with the family, palliative physician at hospital, - Multiface screen function allowed for other family member or HCPs to   join   - Synchronous delivery | - NR |
| Weaver et al., 2020 [55] | To allow for continuity of  pediatric-specific palliative care, while introducing the child and family to their new in-home local home hospice provider | NR | Family, HCPs (palliative physician, home hospice nurse) | - Virtual transition model using FaceTime: in person visit from home hospice nurse, pediatric palliative care physician joined by screen within 48 h of arrival home after hospital discharge - Same intervention occurred on day 14 - Synchronous delivery | - FaceTime |
| Weaver et al., 2021 [56] | To extend care reach into  rural regions and to enable end-of-life care to occur at home | NR | Families, HCPs (home hospice nurse, hospital pediatric palliative care clinician) | - Videoscreen interactions (Zoom Videoconferencing or Facetime platforms) between hospital-based pediatric palliative care clinician and home-based hospice nurse with the family present - Video interactions at minimum every 14-days - Synchronous delivery | - Zoom or facetime platform |
