# Supplementary material for: Health Technologies and Infrastructures for Supporting Home-Based Pediatric Palliative Care: Scoping Review
Source: J Med Internet Res. 2025 Dec 8;27:e70687. doi: 10.2196/70687 (PMC12723365; doi:10.2196/70687)
Supplement: Multimedia Appendix 5 [file jmir_v27i1e70687_app5.docx]

**Multimedia Appendix 5** Characteristics of the included publications

| **Author, year, country** | **Type of literature** | **Aim of study** | **Sample** | **Research design** | **Results** |
| --- | --- | --- | --- | --- | --- |
| Abstracts | | | | | |
| Canter, 2019  The United States (US) | Abstract for conference | To present study design,  including detailed information about Electronic Surviving  Cancer Competently Intervention Program (eSCCIP), along with information about design and implementation of this community‐oriented pilot test | Approximately 30 families (parents of children with cancer, age of children not reported (NR)) | Pilot test | Not applicable (NA) |
| Castro et al., 2021 [69]  Colombia | Abstract for conference | To develop an adapted home monitoring program in children with congenital heart disease | 17 children with congenital heart disease (age NR) | Not reported (NR) | The program has done 693 follow-up calls, 291 video-calls and allowed the identification of 1345 alarms, which led to audio/video calls contacts and informative, educational or clinical recommendations. 8 critical situations prompted emergency consultation and led to in-hospital management including hemodynamic procedures |
| Grootenhuis, 2020 [63]  The Netherlands | Abstract for conference | NR | Children with cancer, parents, health-care professionals (HCPs) | NR | Currently 2 studies have been conducted in a Randomized controlled trial (RCT) for the online course for adolescents and parents. Preliminary results show positive outcomes for both courses |
| Hunter et al., 2018 [37]  The US | Abstract for conference | To describe the preliminary efficacy testing of a mobile health intervention that aims to manage cancer pain and symptoms in children | 60 children with cancer will be included. Present data on 19 children (8-18 years old) completed trial | RCT | The children completed an average of 96.8 diaries over the study period. The intervention group (IG) (*n*=8) experienced lower overall pain severity, average pain since last diary entry and worst pain since last diary entry) compared to the control group (CG) (*n*=11). Pain (31%), trouble sleeping (24%), and vomiting (23%) were the three most common symptoms reported |
| Marc-Aurele et al., 2020 [51]  The US | Abstract for conference | To describe the logistics of using telemedicine (TM) through an outpatient pediatric palliative clinic and to describe the experience of using TM from the perspective of the parent, nurse, and physician | Children with complex chronic conditions (number of children NR, 10 months - 19 years old), family, HCPs | NA | Reason for TM visits included patient comfort, distance from clinic, and aggressive behavior |
| Senguttuvan et al., 2022 [68]  India | Abstract for e-poster | To assess parental satisfaction with tele consultation (TC) services provided during the COVID-19 pandemic | 66 children with cancer and 17 with solid tumor (2-14 years old) from 83 families. | Cross-sectional study | 71% of the participants did not encounter any difficulty in contacting the doctors by TC. Most families expressed ease in communicating problems by TC, were satisfied with the time (81/83), explanation (82/83), thoroughness (82/83), and courtesy (78/83) extended to them during TC. The positive impact of TC was: time-saving (93%), cost-saving (87%), decreased physical exertion (82%), and availability of daily access to care providers (63%). Nearly 75% of the respondents concurred to TC being as good as face-to-face consultation and wanted TC to be a part of routine care |
| Simon et al., 2021 [45]  Canada | Abstract for e-poster | To describe the results of a project to study barriers and facilitators for future implementation of the Pain Monitor app, guided by the Knowledge-to-Action Cycle | 27 families (age range child NR, mean age 7.3 years old), 6 HCPs | Mixed-method part study of a feasibility study | Barriers most often mentioned by families related to specific functionalities of the app (i.e. daily reminders not received consistently, the wish for an overview of previously reported pain scores) and unclear instructions on when app could be used (not during hospitalization). Family app use was facilitated by user friendliness of the app and perceptions it improved care for patients. HCPs most often mentioned time consumption and increased workload as barriers, and improved care for patients as facilitators. Based on the identified barriers and facilitators, the app was optimized to the specific context and wishes of end-users |
| Brief report | | | | | |
| Archer et al., 2021 [73]  The United Kingdom (UK) | Brief report | To identify and synthesize the literature exploring the impact of all digital health interventions on the psychological outcomes of patients and families receiving pediatric palliative care (PPC) | 3 prospective longitudinal studies reported in three papers | Systematic review and narrative synthesis | One study reported no differences in quality of life (QOL) between caregivers in control and intervention groups over a 10-week period, one reported an increased sense of identity and peace of mind in pediatric patients following two sequential digital health visits, and lastly one showed a significant improvement in family empowerment over a 3-month period and increased feelings of control |
| Dreher et al., 2023 [36]  Austria | Brief report | To determine the potential reduction in treatment burden through the expansion of virtual care among children with leukemia | 152 children with leukemia (≤18 years old at diagnosis) | Retrospective cost analysis | Patients living in urban areas traveled median distances of 1555 km compared with 7536 km for patients living in rural areas. For the latter group, a median reduction in travel distance of 3560 km, travel time of 51 hours and CO2 emissions of 623 kg was estimated, if every second visit was replaced by video consultations |
| Weaver et al., 2020 [54] The US | Brief report | To learn from the experiences of rural hospice nurses caring for children at the end of life using telehealth modalities to inform palliative communication | 15 nurses | Type of qualitative design NR | Nurses recommended individualizing communication, pacing content, fostering human connection, and developing relationships even with technology use |
| Case report | | | | | |
| Ellis & Lindley, 2020 [49]  Scotland | Case report | To describe a pediatric hospice provider in Scotland and their experience implementing a telehospice program in response to COVID-19 | A pediatric hospice provider in Scotland | Case report | The organization evaluated its pediatric clinical and wrap-around hospice services and rapidly migrated them to a virtual environment. They creatively added new services to meet the unique needs of the entire family, who were caring for a child at end of life during COVID-19. Their experience highlights the planning and implementing processes of telehospice with key lessons learned, while acknowledging the challenges inherent in using technology to deliver hospice care |
| McLeod & Star 2021 [72]  Australia | Case report | To explore the development and provision of interdisciplinary music therapy and child life therapy telehealth sessions in a tertiary hospital PPC service | 3 children with congenital heart disease, autism  spectrum disorder, spinal muscular atrophy or lafora progressive myoclonus epilepsy (4, 5 and 17 years old) | Case report | These cases highlight that interdisciplinary telehealth sessions in PPC can promote well-being through enjoyable and engaging activities for patients and families, develop parental skill and confidence in playing with their child, and provide opportunity for more frequent sessions that are time efficient for therapists and families |
| Meryk et al., 2021 [41]  Austria | Case report | To present a anecdotal case presented and future challenges in facilitating the success and sustainability of interventions by healthcare providers | A 10-year-old male patient with Burkitt leukemia | Case report | This case highlighted how electronic patient-reported outcome measures can directly facilitate patient care in real time and might be incorporated in future clinical routine |
| Empirical paper | | | | | |
| Bird et al., 2022 [48]  Canada | Empirical paper | To assess areas of strength and opportunity within the DigiComp Kids system, a hospital-to-home intervention for children with medical complexity and their families and care providers | 15 participants (5 hospital-based HCPs, 6 family members, and 4 home-based HCPs) | Usability testing using multi-methods | Participants stressed the need to find the right fit between user needs and the effort required to use the system. Interviews also revealed that the value of the DigiComp Kids system was in its ability to create a digital bridge between hospital and home, enabling participants to foster and maintain connections across boundaries |
| Canter et al., 2019  The US | Empirical paper | To describe a four-phase, mixed-methods, user-centered approach to the design and evaluation of a novel psychosocial intervention for parents of children with cancer eSCCIP, adapted from an in-person intervention (Surviving Cancer Competently Intervention Program) | 14 parents to children with cancer (age of children NR)  Phase 1: 17 parents (from 10 families)  Phase 2: 17 parents (from 10 families)  Phase 3: 5 parents (from 4 families)  Phase 4: 4 parents (from 3 families) | Participatory intervention development process | Initial results suggest that eSCCIP may be an acceptable, feasible, and usable intervention for parents of children with cancer. eSCCIP is now ready for a larger pilot evaluation of usability, feasibility, and impact on target outcomes, including the reduction of anxiety and post-traumatic stress syndrome and improvements in family functioning |
| Canter et al., 2021  The US | Empirical paper | To establish evidence of eSCCIP feasibility, acceptability, and accessibility in a community setting and to evaluate efficacy for key psychosocial outcomes | 29 parents of children with cancer (eligible if caregiver for child 0-17 years old) | Pilot study | Parents rated eSCCIP as highly acceptable, feasible, and accessible. A large clinical effect was detected for acute distress. Moderate clinical effects were reported for overall posttraumatic stress disorder symptoms, negative mood/cognitions, and symptoms of anxiety |
| Foster et al., 2021 [71]  The US | Empirical paper | To evaluate the acceptability, feasibility, and added value of integrating e home monitoring using digital platforms (teleIHM) with synchronous TM video visits and asynchronous video/photo sharing during the interstage period | 41 infants with single ventricle physiology during the interstage period (age range NR, median age 42 days),  18 families | Descriptive program evaluation | A total of 551 VVs were conducted with a median 12 video visits per patient. Parents sent a median 2 pictures. Parents reported an adjustment period to teleIHM but engaged favorably with TM overall. Families felt reassured by the oversight routine TM provided and identified logistical and clinical value to video visits above teleIHM alone, while acknowledging trade-offs with in-person care |
| Jibb et al., 2018 [39]  Canada | Empirical paper | To elucidate the perceptions of adolescents with cancer to determine the acceptability and perceived helpfulness of Pain Squad+, suggestions for app improvement, and satisfaction with the pilot study protocol | 20 adolescents with cancer (12-18 years) | Phenomenological inquiry | Adolescents with cancer generally liked the app and considered it helpful. They reported that the app was easy to use and understand, supported the self-care of pain, and simplified patient-provider communication. The suitability of the app to adolescents’ lives and the acceptability of participating in the pilot were also shown |
| LeBlanc et al., 2024 [58]  The US | Empirical paper | To evaluate the benefits of mobile health monitoring using the KidsHeart app in an infant congenital heart disease (CHD) population | 69 infants with CHD (median age 28 days old) | Pilot study | Subjects submitted 5700 mobile health red flag notifications including 245 violations with 80% (55/69) of subjects submitting at least one violation. Violations precipitated 116 interventions including hospital admission in 34 (29%) with trans-catheter evaluation in 15 (13%) of those |
| Liu et al., 2022 [41]  China | Empirical paper | To develop a WeChat Mini Program based on previous studies to satisfy the needs of parents of children with cancer and encourage them to participate in symptom management, making it easier for family-centered care | 10 parents of children with cancer, children with cancer (aged 5–17 years old), 15 experts in pediatric oncology and informatics (9 experts in the field of clinical nursing of childhood  cancer, 2 experts in cancer nursing research, 2 doctors in the field of  childhood cancer, and 2 IT specialists) and 10 pairs of parents and children with cancer (aged 5-17 years old) | Multi-methods (interview, survey) | Most users reported that the Mini Program is user-friendly, with satisfaction scores on the Poststudy System Usability Questionnaire ranging from 5.2 to 5.7 (out of 7.0) in four dimensions. Most participants thought the Mini Program was convenient, easy to use, and helpful |
| Mehdizadeh et al., 2022 [64]  Iran | Empirical paper | To develop and preliminary evaluate a cancer self-management system (CanSelfMan) tailored to the needs of children with cancer and their parents/ caregivers | 8 children with cancer (age 7-14 years old), 10 parents, multidisciplinary team (software engineers, medical informatics specialists, oncologists, pediatricians, psychologists) | User-centered design approach | The CanSelfMan app can support these groups by providing access to reliable information about cancer, facilitating communication between children or parents and HCPs, and helping promote medication adherence through a reminder function |
| Mehdizadeh et al., 2023 [65]  Iran | Empirical paper | To evaluate the usability of CanSelfMan, a self-management app that provides access to reliable information to improve communication between HCPs and children with cancer and their parents/caregivers, facilitating remote monitoring and promoting medication adherence | 19 children with cancer (7-14 years old) and 25 parents/caregivers | Usability and compatibility study | According to the children’s evaluations, attractiveness and efficiency achieved the best mean results compared with novelty. Parents/caregivers rated efficiency at a mean of 1.880 and attractiveness at a mean of 1.853. The lowest mean score was reported for novelty |
| Meryk et al., 2025 [57]  Austria | Empirical paper | To evaluate the feasibility of using synchronous telemedicine for routine clinical care during the maintenance therapy phase | 22 children with acute leukemia; 5 children regular cancer care, 17 children TM-centered cancer care (3-7 years old) | Cohort study | Rural patients had a total of 510 routine clinical visits, 72% conducted through TM. Patients' health-related quality of life remained within the normative range, and user satisfaction with the TM was notably high. The implementation of the TM resulted in savings of 70,158 km, 950 h of travel, and 12,277 kg CO_2_ emissions |
| Meryk et al., 2021 [42]  Austria | Empirical paper | To evaluate the feasibility and value of daily patient-reported outcome measures by children receiving chemotherapy for cancer | 12 children with cancer (5-18 years old) | Prospective,  single-arm longitudinal study | Severe symptoms were reported in 14.7% of measurement time points, which led to prompt health care interventions in 57 cases, including extension of supportive care and pre-emptive inpatient admissions. Over 80% of the patients and their proxies provided feedback with high rating for satisfaction and usefulness of ePROtect |
| Munoz-Bonet et al., 2020 [50]  Spain | Empirical paper | To assess the usefulness of TM in the reduction of hospital confinement among ventilator dependent children by achieving early and, ideally, permanent discharge to home care, with the familial, social and financial benefits that this implies, but without affecting quality of care | 12 tracheo-stomized ventilator-dependent children (aged < 16 years old) and their families | Prospective clinical study | 11 events required hospitalization (7.8%) but 38 (27.0%) hospitalizations were avoided. The emergency readmission time accounted for 0.99% of the total time. 6 patients were decannulated, and one patient died due to primary cardiac arrest. All families considered that TM had helped to avoid hospital visits, was not an intrusion into their privacy, and improved the child’s safety and QOL |
| Novrianda et al., 2023 [44]  Indonesia | Empirical paper | To develop and trial the Chemo Assist for Children mHealth app for symptom management in children with acute lymphoblastic leukemia | 31 parents of children with acute lymphoblastic leukemia (0-18 years old)  10 parents of children with acute lymphoblastic leukemia (2-13 years old) | User-centered design | The app mHealth was a valid, accessible, and appropriate app for users |
| Phipps et al., 2020 [66]  The US | Empirical paper | To evaluate whether a web-based version of Bright IDEAS requiring fewer resources is noninferior to in-person administration | 621 caregivers of children with newly diagnosed cancer (child age range NR, mean age child web-based Bright IDEAS. 8.3 (standard deviation (SD) 5.5), mean age child standard bright 8.2 (SD 5.5)) | Multicenter, RCT with a noninferiority design | Parents in the web-based Bright IDEAS group improved their problem-solving, but the web-based intervention preserved 60% of the standard treatment effect, the test of noninferiority was non-significant. The web-based intervention preserved > 60% of the standard intervention effect on all secondary outcomes. Tests of noninferiority were non-significant |
| Schultz et al., 2021 [70]  The US | Empirical paper | To examine whether sociodemographic and clinical care variables are associated with portal activation in a pediatric oncology sample | 258 caregivers to 390 children with cancer (age range NR, mean age 8.2 SD 5.5) | Retrospective cross-sectional chart review | Caregivers with a younger child, spoke English, lived closer to the hospital, lived in higher child opportunity index area, with longer treatment length, and more radiology tests had greater odds of portal activation. Those with private health insurance or white race were overrepresented among those who activated an account in univariate analysis |
| Simon et al., 2021 [46]  The Netherlands | Empirical paper | To assess adherence to, feasibility of, and barriers and facilitators of implementation of an app developed to monitor and follow-up with pain in children with cancer at home | 27 children with cancer (age 1-17 years old), 6 HCPs | Feasibility study using mixed methods | 63% (N=17) of families used the app daily for 3 weeks, and 18.5% (N=5) reported pain scores twice daily during that time (family adherence). 12 out of 27 children (44.4%) reported a clinically significant pain score at least once. In 70% (14/20) of clinically significant pain scores, HCPs followed-up with families within the set timeframe (HCP adherence). Outcomes reveal feasibility for most app functions (i.e., positive evaluation by ≥ 70% families/ HCPs), and non-feasible aspects could be resolved |
| Simon et al. 2024 [47]  The Netherlands | Empirical paper | To compare pain monitoring app in a home setting to a  control group to assess whether use of the app yielded a lower prevalence of clinically significant pain in children with cancer at home (aim 1). Secondary, to compare pain severity, duration, interference, pain management strategies, and parental emotional well‐being between the two groups (aim 2). Finally, to evaluate the use, acceptability and relevance of the app with children and parents in the intervention group (aim 3) | 158 children with cancer (age 0-18 years old). 79 children in the intervention group and 79 in the control group completed the study | RCT | The app group reported significantly  less clinically significant pain and significantly lower pain severity compared to usual care. No differences were found for duration, interference, or management strategies. Parents in the app group reported significantly less distress compared to usual care. Families generally evaluated the app positively |
| Stagg et al., 2023 [52]  The US | Empirical paper | To investigate adding TM with synchronous video visits to the Infant Single Ventricle Monitoring Program | 29 infants with hypoplastic  left heart syndrome | Pilot study | Five TM visits led to expedited outpatient assessments, of which 1 patient required hospitalization. There were no missed events or deaths. Median emergency department visits/patient/month were significantly lower compared to the same calendar period of the prior year. Caregivers and clinicians expressed high levels of satisfaction with TM. TM for this high-risk population is feasible and effective in identifying clinical concerns and preventing unnecessary emergency department visits |
| Weaver et al., 2020 [55]  The US | Empirical paper | To determine whether telehealth inclusion of a familiar PPC provider during the first two home-based hospice visits was acceptable to children, families, and adult-trained home hospice nurses in rural settings | 15 children in need of home-based palliative care (age (6 months - 15 years old), 15 family caregivers (11 mothers, 2 grandmothers, 2 fathers), 15 nurses | Case series | Home distance averaged 172 miles with mean 8 hours saved by accessing telehealth encounter. Visit content was primarily caregiver support, QOL, goals of care, symptom management, and medication review. Telehealth acceptability improved between time points and was higher in family caregivers than hospice nurses. All children able to self-report stated a ‘‘like’’ for telehealth, citing 6 reasons such as ‘‘being remembered’’ and ‘‘medical knowledge and care planning |
| Weaver et al., 2021 [56]  The US | Empirical paper | To explore physical and emotional symptom burden and family impact assessments for children with terminal cancer receiving home based-hospice care | 11 children with cancer | Pilot study | PPC telehealth combined with adult-trained rural hospice providers may be utilized to support pediatric oncology patients and their family caregivers as part of longitudinal home-based hospice care |
| Protocol paper | | | | | |
| Bradford et al., 2021 [34]  Australia | Protocol paper | To evaluate: (1) the effectiveness of electronic patient reported outcome measures to generate stratified alerts, symptom management recommendations and graphical summaries (the RESPONSE system) to improve health outcomes and (2) the implementation of the RESPONSE system by assessing feasibility, acceptability, satisfaction, and sustainability | Children receiving active treatment for blood cancer or solid tumors (aged 4- 18 years old), their caregiver(s) will be included | A pragmatic hybrid effectiveness-implementation controlled trial, using mixed methods | NA |
| Canter et al., 2023  The US | Protocol paper | To test eSCCIP/ El Programa Electronico de Intervencion para Superar Cancer Competentemente (eSCCIP-SP) in a multisite RCT, compared to an internet-based education control condition consisting of information specifically focused on concerns relevant to parents and caregivers of children with cancer | 350 eligible parents and caregivers of children with cancer (aged 0-18 years old) will be recruited | RCT | NA |
| Jibb et al., 2020 [38]  Canada | Protocol paper | To longitudinally evaluate the impact of Pain Squad+, with or without the addition of nurse support, on adolescent health and cost outcomes | 74 adolescent (12-18 years old) with cancer per arm will be included | Pragmatic, multicenter, waitlist controlled, 3-arm parallel-group superiority RCT | NA |
| Lai et al., 2023 [40]  The US | Protocol paper | To examine the effects of the SyMon-SAYS system on perceived barriers to symptom management, self-efficacy, and symptom severity | 200 children with cancer (aged 8-17 years old) and their parents, HCPs (physicians, fellows, nurse practitioners, and nurses) will be recruited | Single-institution waitlist RCT | NA |
| Sengul & Toruner, 2020 [67]  Turkey | Protocol paper | To examine the impact of a technology‑based psychosocial motivation program on children and families who are being followed up on a diagnosis of cancer | Children with leukemia (aged 9–18 years old) and their parents will be recruited | RCT | NA |
| Warniment et al., 2023 [53]  The US | Protocol paper | To describe how to compare the effectiveness of the “Garnering effective telehealth 2 help optimize multidisciplinary team engagement” (GET2HOME)  transition bundle intervention to the standard hospital‐based care coordination discharge process by assessing healthcare reutilization and patient‐ and family‐centered outcomes | Children and young adults with medical complexity | Pragmatic 2-arm RCT | NA |
| Review paper | | | | | |
| Holmen et al., 2020 [26]  Norway | Review paper | To identify and review the use of eHealth to communicate and support home-based PPC and appraise the methodological quality of the published research | 2 qualitative studies, 1, case study, 1 case-control study, 1 cost-minimization analysis, 1 longitudinal, multisite mixed methods study, 1 non-randomized pilot study | Convergent, systematic mixed methods review | eHealth to facilitate remote pediatric palliative care was acknowledged both as an intrusion and as a support at home |
| Brock et al., 2018 [35]  United States | Review paper | To describe emerging methods of symptom and health-related QOL (HRQOL) assessment through patient-reported outcomes (PROs) tools now used in clinical practice and novel research studies | NA | Review without methods | Computer-based and mobile apps can facilitate assessment of symptoms and HRQOL. These technologies can be used alone or combined with therapeutic strategies to improve symptoms and coping skills |
